# Lubricious anti-adhesive interface prevents friction, biofilm, and encrustation in long-term indwelling ureteral stents

**DOI:** 10.1016/j.mtbio.2026.103405

**Published:** 2026-06-29

**Authors:** Yejin Jo, Yeontaek Lee, Sungun Bang, Kayoung Son, Kijun Park, Dokyun Kim, Soo A Kim, Seonghyeon Eom, Inhee Choi, Su-Jin Shin, Kyo Chul Koo, Jungmok Seo

**Affiliations:** a School of Electrical and Electronic Engineering, Yonsei University, Seoul, 03722, Republic of Korea; b Department of Urology, Gangnam Severance Hospital, Yonsei University College of Medicine, Seoul, 06273, Republic of Korea; c Division of Engineering in Medicine, Department of Medicine, Brigham and Women's Hospital, Harvard Medical School, MA, 02139, USA; d Department of Laboratory Medicine and Research Institute of Bacterial Resistance, Yonsei University College of Medicine, Seoul, 06273, Republic of Korea; e Department of Life Science, University of Seoul, Seoul, 02504, Republic of Korea; f Department of Applied Chemistry, University of Seoul, Seoul, 02504, Republic of Korea; g Department of Pathology, Gangnam Severance Hospital, Yonsei University College of Medicine, Seoul, 06273, Republic of Korea

**Keywords:** Ureteral stents, Lubricant reservoirs, Lubricant-infused interfaces, Low-friction, Anti-biofouling, Long-term encrustation resistance

## Abstract

Ureteral stents are essential for relieving urinary obstruction and preserving renal function. However, long-term indwelling often induces friction-mediated urothelial irritation, bacterial colonization, and mineral encrustation, which impair urinary drainage and complicate stent removal. Here, we developed a friction-lowering urinary interface for defense against encrustation (FLUID) by integrating a silicone-based primer and lubricant layer into a fully microporous thermoplastic polyurethane framework. Unlike conventional slippery liquid-infused porous surfaces that retain lubricant primarily within surface microstructures, the fully microporous TPU framework serves as an internal lubricant reservoir throughout the stent wall, replenishing the lubricating interface under dynamic urinary conditions. Under sterile conditions the FLUID coating suppressed planktonic bacterial adhesion (*E. coli*, *B. cereus*, *S. aureus*; 72 h adhesion assay) by more than 94% relative to uncoated substrates. In a *P. mirabilis*–spiked 14-day mature biofilm model, biofilm-associated bacterial recovery from FLUID was modestly lower than from the commercialized comparators (Log R ≈ 0.10 and 0.14; approximately 21% and 28% reduction). In a porcine model, the coated stent reduced inflammatory activity and attenuated mineral accumulation after 4 weeks, with magnesium and calcium deposition decreased by 84.1% and 45.1%, respectively. After 8 weeks, scanning electron microscopy confirmed clean surfaces with patent side holes and minimal debris accumulation. Overall, the FLUID stent establishes a self-replenishing lubricant interface integrated throughout the microporous stent wall, enabling simultaneous mitigation of friction, early biofouling, and mineral deposition, and providing a clinically relevant proof-of-concept strategy for safer long-term urinary drainage that will require further preclinical validation prior to clinical adoption.

## Graphical abstract

**Figure:**
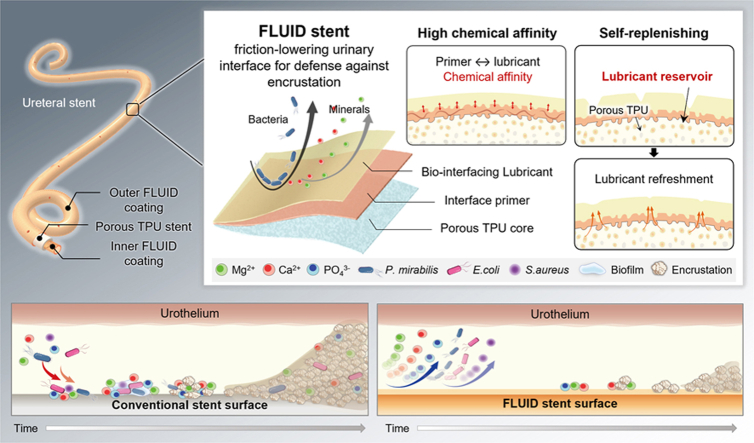

## 1 Introduction

Ureteral stents are widely employed in the management of ureteral obstruction to maintain urinary drainage and preserve renal function [1]. Their clinical indications include urolithiasis, benign or malignant ureteral stricture, postoperative urinary leakage, and perioperative ureteral protection. Since the introduction of the double-J configuration, polymer-based stents have remained the predominant platform because they provide mechanical flexibility, procedural familiarity, and compatibility with routine endourologic placement [[2], [3], [4]]. Despite these practical advantages, long-term indwelling remains clinically challenging. Device-related pain, hematuria, dysuria, urinary tract infection, biofilm formation, and progressive encrustation continue to constrain the safe dwelling period and frequently necessitate early exchange [[5], [6], [7]]. Clinical indwelling is therefore generally limited to 3 to 6 months [8], as prolonged residence markedly increases the risks of infection, encrustation, obstruction, and difficult retrieval. Current management still relies largely on periodic stent replacement and adjunctive antibiotic therapy, neither of which addresses the surface-mediated mechanisms that underlie long-term device failure.

The deterioration of ureteral stent performance is initiated at the implanted interface. During insertion and subsequent residence, repeated contact between the stent surface and the urothelium generates frictional stress that contributes to mucosal irritation and patient discomfort. Following implantation, the surface is immediately exposed to urinary proteins, dissolved salts, and microorganisms. Adsorption of urinary constituents gives rise to an early conditioning layer that facilitates bacterial attachment and subsequent biofilm maturation. This biologically altered interface then promotes the retention and crystallization of inorganic urinary species, including calcium (Ca^2+^), magnesium (Mg^2+^), and phosphate (PO_4_^3−^) ions containing salts, ultimately leading to luminal narrowing, side hole occlusion, impaired drainage, and difficult retrieval [9,10]. The degree of mineral deposition correlates strongly with placement duration, occurring in approximately 26.8% of stents removed within six weeks and in more than 75.9% after twelve weeks [11,12]. This process becomes particularly severe in patients who develop urinary tract infection (UTI), which occurs in approximately 14.8% of those with indwelled stents [13]. Infection acts as a key catalyst for rapid and extensive encrustation [9,[14], [15], [16], [17]]. Among these organisms, *Proteus mirabilis* (*P. mirabilis*) is of particular importance because it hydrolyzes urea to ammonia, induces urinary alkalinization, and strongly promotes infectious encrustation during indwelling. With this mechanism, infected ureteral stents can progress rapidly to obstruction, and approximately half of infected stents may become obstructed within four weeks [18]. These observations indicate that durable ureteral stent performance requires simultaneous mitigation of insertion-related friction, early biofouling, and infection-driven mineral deposition.

Commercial ureteral stents have already incorporated several design strategies intended to improve patient comfort and reduce indwelling complications. Current products use materials that soften at body temperature [17,19,20], highly smooth surfaces with optimized wettability [21], and geometries that favor urinary drainage [22]. Representative commercial examples reflect this development trajectory. Polaris™ Ultra is used as a hydrophilic stent platform that benefits from pre-use hydration to maximize lubricity [23]. Tria™ incorporates PercuShield™ technology as a commercially developed anti-fouling surface chemistry [3]. Inlay Optima™ is designed to maintain an ultra-smooth surface intended to reduce deposition and improve handling [24]. These refinements have improved specific aspects of device performance, yet long-term encrustation remains a persistent challenge. Comparative analyses of urinary biomaterials have shown that clinically used polymeric stents still accumulate substantial deposits over time, and no currently available polymeric platform completely prevents long-term encrustation under variable urinary conditions. Even among commercially optimized devices, mineral accumulation remains strongly influenced by urine chemistry and infection status. The continued need for scheduled exchange indicates that current commercial strategies improve handling and comfort, but do not maintain a sufficiently stable low-fouling surface throughout prolonged urinary exposure.

To further improve ureteral stent performance, a broad range of surface modification strategies has been explored beyond currently available commercial platforms. Antibacterial interfaces have been introduced to suppress microbial colonization and infection associated fouling. A mussel-inspired clickable antibacterial peptide coating improved bacterial resistance and reduced encrustation *in vivo* over an extended period [25], indicating that biologically active surface chemistry can beneficially modulate the urinary response to implanted stents. Hydrophilic polymer coatings have also been pursued, as hydrated surfaces can reduce interfacial resistance through the formation of hydrated boundary layers. A recent polyvinylpyrrolidone and citric acid coating on polyurethane ureteral stents showed improved wettability [26], an approximately four fold reduction in friction coefficient, peel strength up to 600 N/m, and more than 90% suppression of bacterial adhesion, while maintaining reduced encrustation for up to one month in artificial urine. Hydrogel-based lubricious coatings have likewise been investigated to facilitate insertion through pronounced hydrophilicity [27]. These studies show that hydrated and functional coatings can improve selected aspects of ureteral stent performance. However, important limitations remain under prolonged urinary exposure. Antibacterial coatings are primarily directed toward microbial control and may not sufficiently alleviate insertion-related friction or chronic urothelial irritation. Hydrated polymer and hydrogel layers can also remain susceptible to swelling, surface damage, delamination, or gradual functional decline under repetitive abrasion and prolonged aqueous exposure. Consequently, low-friction insertion, resistance to biofouling and mineral deposition, and long-term interfacial stability are not easily maintained within a single surface platform during extended stent indwelling.

Lubricant-mediated anti-adhesive interfaces have attracted increasing interest as an interfacial strategy capable of simultaneously reducing friction and suppressing stable attachment of proteins, cells, and microorganisms [[28], [29], [30], [31], [32], [33], [34], [35]]. Slippery liquid-infused porous surfaces represent a prominent example of this approach, in which a mobile lubricant layer minimizes direct solid contact while maintaining a low shear boundary [36]. However, conventional SLIPS systems typically rely on lithographically defined, templated, or etched surface microstructures that confine the lubricant within a thin superficial layer, rendering them vulnerable to progressive depletion under sustained hydrodynamic shear and repeated mechanical contact. Moreover, reproducing such delicate surface patterns uniformly over long, narrow, curved polymeric devices has been challenging, and the vast majority of SLIPS biomedical studies have therefore been performed on planar substrates or short tubing segments rather than full-length implantable devices. Additional state-of-the-art anti-fouling strategies, including tethered liquid perfluorocarbon interfaces, zwitterionic polymer brushes, and antimicrobial peptide coatings, have likewise been explored in biomedical contexts (Supplementary Table 1). These systems have each demonstrated promising anti-fouling performance, but their translation to full-length, clinically usable ureteral stents has remained limited by combinations of fluorinated chemistry, fabrication complexity, coating durability, and the absence of long-term *in vivo* validation. These fabrication and durability constraints, together with increasing interest in non-fluorinated material platforms for implantable devices, remain major barriers to clinical implementation.

In this work, we developed the friction-lowering urinary interface for defense against encrustation (FLUID) by integrating a primer lubricant coating system with a fully microporous thermoplastic polyurethane (TPU) double-J stent platform in which microporosity extends throughout the entire stent wall. In this architecture, chemical affinity between the primer and lubricant stabilizes interfacial anchoring, whereas the wall-spanning microporous TPU framework functions as a distributed internal lubricant reservoir, thereby supporting sustained retention and continuous self-replenishment during prolonged exposure and local depletion. This material-integrated architecture extends the lubrication concept of slippery liquid-infused surfaces to a clinically relevant tubular device while avoiding complex nanofabrication and remaining compatible with a simple dip coating process suitable for batch production and scale-up. FLUID also addresses a practical limitation of current hydrophilic commercial stents. In our friction comparison, conventional controls were pre-soaked in phosphate buffered saline for 10 min to maximize lubricity, whereas FLUID exhibited the lowest friction without any pre-hydration step. Relative to commercial double-J stents, FLUID reduced pull-out force to 0.07 ± 0.02 N, corresponding to a 7.9-fold reduction versus Polaris™ Ultra and an approximately four fold reduction versus Tria™ and Inlay Optima™. The coating maintained low friction after 30 repeated rubbing cycles, suppressed protein deposition by 93%, and reduced bacterial adhesion by 94.2% relative to uncoated substrates. In artificial urine, FLUID reduced calcium and magnesium deposition after 2 weeks, and in a porcine implantation model it markedly decreased inflammatory activity and mineral accumulation after 4 weeks, with magnesium and calcium deposition reduced by 84.1% and 45.1%, respectively. Scanning electron microscopy at 8 weeks further revealed clean surfaces with patent side holes and minimal debris retention. These results establish FLUID as a lubricant-based ureteral stent platform that integrates a wall-distributed lubricant reservoir, durable interfacial lubrication, scalable coating implementation, and immediate procedural usability without a pre-hydration step.

## 2 Experimental section

### 2.1 Fabrication of the FLUID stent

The stent core was extruded using Pellethane® TPU with foaming agent. Porosity was measured using a mercury intrusion porosimeter (PM33GT, Quantachrome). The stents were cut into 0.5 cm segments and loaded into a cylindrical sample cell (1 cm diameter, 3 cm height) for analysis. The substrate was dip coated in an interface primer solution (Lynksolution B1, Lynk Solutec Inc., Korea) using a dip coater with a withdrawal speed of 130 mm min^−1^. Vacuum was applied in a desiccator during coating to facilitate penetration of the primer into the inner lumen and porous structure of the stent. The coated samples were cured at room temperature for 24 h. The bio-interfacing lubricant (Lynksolution B2, Lynk Solutec Inc., Korea) was then applied by dip coating under the same coating conditions. After coating, the stents were positioned vertically (90°) to drain excess lubricant from the surface and lumen. Biocompatibility was tested on our lubricant in accordance with ISO 10993 standards, including cytotoxicity, pyrogenicity, intracutaneous reactivity, and acute systemic toxicity. All tests met the required acceptance criteria, supporting the preliminary biocompatibility of the material for medical device applications.

### 2.2 Interfacial friction measurement

Rectangular specimens (2 × 6 cm) were prepared from thermoplastic polyurethane (TPU) substrates. The FLUID coating was applied using the dip-coating procedure described above and cured under ambient conditions. Interfacial friction was evaluated using a custom linear sliding fixture designed to reproduce the contact geometry between the coated surface and a polycarbonate counterface. Tangential friction force was monitored using a calibrated commercial load cell mounted on a motorized translation stage. Five independent specimens were tested for each condition. The measurement configuration was adapted from the principles of ASTM D1894 and ISO 8295. Measurements were performed at a sliding speed of 10 mm min−1 under a constant normal load of 0.5 N. The kinetic coefficient of friction was calculated as μ_k_ = F_k_/F_N_, where F_k_ is the average steady-state tangential force and F_N_ is the applied normal load. The initial transient peak was excluded from the calculation.

### 2.3 Surface characterization of FLUID coating

The chemical compositions of the coating were analyzed using x-ray and ultraviolet photoelectron spectroscopy system (XPS-UPS) (AXIS Supra+, Kratos Analytical, UK) equipped with an Al/Ag dual X-ray source. The cross-sectional morphologies of the stent were examined using field-emission scanning electron microscope (FE-SEM) and chemical composition was analyzed using energy-dispersive X-ray spectroscopy (EDS) equipped with SEM (IT-500HR, JEOL, Japan). The fluorescence bio-interface lubricant image was examined using a laser scanning confocal microscope (LSM 900, Carl Zeiss, Germany) at specific excitation/emission wavelengths corresponding to the fluorescent label of coumarin 6. Contact angle (CA) and sliding angle (SA) were measured using a biofluid measurement system equipped with a dynamic image capture camera (Smart Drop, FEMTOBIOMED, Korea). The CA was conducted with 5 μL droplets and the SA was conducted with 10 μL of various biofluids including horse blood (Horse blood Defibrinated, Kisanbio, Korea), artificial mucus (Artificial nasopharyngeal fluid, TMABio, Korea), simulated urine (Simulated urine normal, Biozoa, Korea), phosphate-buffered saline (PBS), and DI water. Blood staining test was prepared with 76 × 26 mm sized uncoated and FLUID-coated TPU. The substrates were tilted at a 60° angle, and 50 μL of horse blood was carefully dropped onto the surface from a height of ≈1 cm.

### 2.4 *In vitro* biocompatibility assessment

To evaluate cell viability and morphological responses, 6 Fr stents were cut into 5 mm segments and used as test substrates, either unmodified or coated with interface primer and FLUID. A transwell culture system (pore size: 8 μm, Corning Inc.) was employed to allow indirect exposure of cells to the stent segments. NIH-3T3 fibroblasts were seeded at a density of 0.5 × 10^5^ cells/mL in 6-well plates containing 1.5 mL of high-glucose Dulbecco's Modified Eagle's Medium (DMEM, 11965-092, Gibco, USA) supplemented with 10% fetal bovine serum (FBS, A5670701, Gibco, USA) and 1% penicillin-streptomycin (15-070-063, Gibco, USA). Cell viability was assessed using a Live/Dead viability kit (L3224, Invitrogen, USA) according to the manufacturer's instructions, and fluorescence images were acquired at 10× magnification with an inverted fluorescence microscope (IX81, Olympus, Japan). Quantitative analysis of relative fluorescence intensity was performed to compare viability after 1, 3, and 5 days of culture. In parallel, metabolic activity was evaluated using the cell counting kit-8 (CCK-8, Thermo Scientific, Pittsburgh, PA, USA). At each time point (1, 3, and 5 days), 100 μL of CCK-8 reagent was added per well and incubated at 37 °C for 2 h. Absorbance at 450 nm was measured using a microplate reader (Synergy H1, Biotek, USA), and relative viability was calculated. For morphological assessment, the cell aspect ratio (major/minor axis) was quantified. Cytoskeletal organization was visualized by staining with Alexa 594-conjugated phalloidin (Thermo Scientific, Pittsburgh, PA, USA), and nuclei were counterstained with DAPI (Thermo Scientific, Pittsburgh, PA, USA). Morphological imaging was conducted using a confocal microscope, and images were analyzed with ImageJ/FIJI software.

### 2.5 Assessment of stability in urine

Urine fouling tests were performed using 5 cm segments of uncoated and FLUID stents. The samples were immersed for 5 min, then longitudinally bisected to allow evaluation of both outer and inner luminal surfaces. The pH of the artificial urine was adjusted in the range of 4 to 9 using 0.1 M hydrochloric acid (HCl, Sigma-Aldrich, USA) and 0.1 M sodium hydroxide (NaOH, Sigma-Aldrich, USA). The pH values were confirmed with a calibrated pH meter. The uncoated and FLUID-coated samples were immersed in these solutions for 7 days, after which the wettability was evaluated by measuring the water contact angle in deionized water.

### 2.6 Mechanical robustness test of FLUID coating

A fluorescently doped lubricant was prepared by dissolving coumarin 6 at a concentration of 1 mg/mL followed by vortex mixing and syringe filtration to ensure homogeneous dye dispersion. The long-term stability test was conducted by uniformly applying the labeled lubricant onto the substrate surface and subjecting the samples to continuous orbital shaking at 100 rpm for four weeks to simulate a mechanically aggressive environment. After incubation fluorescence intensity was quantitatively measured using a fluorescence spectrometer (FS5, Edinburgh Instruments, UK) over the 400–600 nm wavelength range to assess lubricant retention under dynamic conditions. The abrasion test was performed by coating the substrate with the dyed lubricant and monitoring lubricant distribution using real time confocal laser scanning microscopy. Localized mechanical disturbance was applied using a cotton swab to evaluate lubricant depletion and subsequent spontaneous replenishment behavior. The surface area coverage of the lubricant was quantitatively analyzed using ImageJ/FIJI software. To evaluate lubricant retention under confined dynamic conditions, a 5 cm stent was inserted into an artificial ureter and placed in a conical tube containing artificial urine, followed by 1000 cycles of shaking. At each time point, aliquots of the artificial urine were collected and the fluorescence intensity of released lubricant was measured using a microplate reader (Synergy H1, Biotek, USA). Residual lubricant on the stent surface was visualized using a laser scanning confocal microscope (LSM 900, Carl Zeiss, Germany). The surface area coverage of the lubricant was quantitatively analyzed using ImageJ/FIJI software.

### 2.7 Stent migration test

The stent migration tests were conducted using 10 cm segments of uncoated and FLUID stents with one pig tail end. The stents were inserted into an artificial ureter (Aldaver, Korea), and the opposite straight end was pulled through to evaluate resistance to migration. The test was performed using a mechanical testing machine (MultiTest 2.5-DV, Mecmesin, UK) equipped with a 50 N load cell at a crosshead speed of 30 mm/min.

### 2.8 Mechanical friction and lubricant retention under repetitive contact

Friction tests were conducted using 10 cm segments of uncoated and FLUID-coated stents. Conventional stents including Tria™ (Boston Scientific, USA), Polaris™ Ultra (Boston Scientific, USA), and Inlay Optima™ (Bard Medical, USA) were used as control groups. To simulate the ureteral passage, a guide hole was created in an artificial ureter block with a needle, and each stent was advanced through the channel. The frictional resistance during passage was measured using a mechanical testing machine (MultiTest 2.5-DV, Mecmesin, UK) equipped with a 50 N load cell at a crosshead speed of 50 mm/min. Lubricant retention under extended physical friction was evaluated by inserting the coated stent into an artificial ureter and applying 1000 cycles of mechanical rubbing. After the friction cycles, the distribution of the fluorescently labeled lubricant was examined using a laser scanning confocal microscope (LSM 900, Carl Zeiss, Germany). The surface area coverage of the lubricant was quantitatively analyzed using ImageJ/FIJI software.

### 2.9 Protein and bacterial adhesion test

Protein adhesion tests were conducted using TPU samples with a size of 5 mm × 5 mm. Albumin from bovine serum plasma conjugated with fluorescein isothiocyanate (A23015, Invitrogen, USA) was dissolved in PBS to a final concentration of 1 mg/mL. Fibrinogen (Invitrogen, USA) was dissolved in PBS to a final concentration of 150 μg/mL. Pristine and FLUID-coated TPU samples were prehydrated in PBS, immersed in the protein solutions, and incubated at 37 °C for 24 h. After incubation, the samples were rinsed gently with deionized water, and fluorescence images were obtained using an inverted fluorescence microscope (IX81, Olympus, Japan). The images were analyzed with ImageJ/FIJI software. Bacterial adhesion tests were conducted using *Escherichia coli* (*E. coli*, 25922, ATCC, USA), *Bacillus cereus* (*B. cereus,* 11778, ATCC, USA), and *Staphylococcus aureus* (*S. aureus*, 25923, ATCC, USA). Stent samples were immersed in bacterial suspensions and incubated at 37 °C for 72 h. After incubation, the samples were gently rinsed with PBS to remove nonadherent bacteria, and fluorescence images of adherent bacteria were obtained using an inverted fluorescence microscope (IX81, Olympus, Japan).

### 2.10 *In vitro* encrustation assay

*In vitro* encrustation tests were performed at Innovotech Inc. (Edmonton, Canada) using the BEST Assay™ system. Ureteral stent segments (3.5 cm) from uncoated control, Inlay Optima™ (Bard Medical, USA), Tria™ Soft (Boston Scientific, USA), and FLUID stent were aseptically mounted on 12-well BEST Assay™. Wells were filled with 4 mL of artificial urine, and plates were incubated at 37 °C on a rotary shaker (20 ± 10 rpm) for 14 days. The medium was replaced every 48 h with fresh sterile urine. Two models were tested: (i) sterile urine and (ii) urine spiked on day 1 with *P. mirabilis* (∼10^6^ CFU/mL). The inoculum was an authenticated *P. mirabilis* reference strain (ATCC 7002) suspended in sterile artificial urine. The artificial urine was confirmed sterile prior to inoculation, and the 14-day assay was conducted under closed-loop rotation (20 ± 10 rpm) without exposure to ambient microorganisms; bacteria recovered from the stent surface at the assay endpoint are therefore attributable to the inoculated strain. Species-confirmation methods such as MALDI-TOF MS or 16S rRNA sequencing of recovered colonies were not performed in the present study and are identified as a methodological refinement for future infection-model work. After incubation under sterile conditions, samples were rinsed three times in sterile water without agitation and digested in 4% nitric acid. After 30 min sonication, the solutions were left for 24 h before quantification of Ca and Mg by inductively coupled plasma optical emission spectroscopy (ICP-OES). In the infection model, samples were similarly rinsed, then sonicated in 0.05% Tween-80 in PBS for microbial recovery. Aliquots were serially diluted and spot-plated to determine CFU counts. Residual suspensions and stent pieces were subsequently digested in 8% nitric acid for encrustation analysis by ICP-OES. Selected segments were fixed in glutaraldehyde, dehydrated, sputter-coated, and examined by SEM (Zeiss EVO MA10).

### 2.11 *In vivo* preclinical test in porcine model

Yorkshire minipigs (*Sus scrofa* domesticus, Optipharm Inc., Cheongju, Korea) were selected due to their urinary tract anatomy and renal physiology closely resembling those of humans, as well as their relatively slower growth rate, which facilitates long-term observation. Female pigs weighing between 40 and 45 kg were used in all cases to facilitate cystoscopic identification of the ureteral orifices and to minimize the effect of urethral resistance. All experimental procedures were approved by the Yonsei Biomedical Research Ethics Committee after reviewing the study protocol (2023-0262) and are reported in accordance with the Animal Research: Reporting of *In vivo* Experiments (ARRIVE) Guidelines. Animals were fasted 12 h prior to anesthesia. Anesthesia was induced with intramuscular administration of alfaxalone (0.1 mg/kg), medetomidine (0.05 mg/kg), and azaperone (0.05 mg/kg). Orotracheal intubation was performed, and anesthesia was maintained with isoflurane (2.0%). In the lithotomy position, cystoscopy was performed to identify both ureteral orifices. A hydrophilic safety guidewire (ZIPwire; Boston Scientific, Marlborough, MA, USA) was advanced into the collecting system. To minimize inter-animal variability, each pig received a conventional stent (Tria™, Boston Scientific, Marlborough, MA, USA) in the left ureter and a FLUID-coated stent in the contralateral right ureter, enabling paired within-subject comparisons. The side assignment (conventional stent, left; FLUID, right) was fixed rather than randomized, and potential side-specific anatomical effects therefore cannot be fully excluded. All stents were soaked in saline before use, without lubricants. Proper placement was confirmed by fluoroscopic and cystoscopic imaging immediately after placement. At 4 and 8 weeks, stents were retrieved transurethrally using a cystoscope and forceps. This observation period was determined based on ethical guidelines and institutional animal care standards, prioritizing humane endpoints.

Prior to removal, the degree of ureteral orifice inflammation was evaluated cystoscopically. To assess the effect of each stent on the ureteral mucosa, ureteral specimens were harvested *in vivo* using laparoscopic instruments. A 1 cm mid-ureteral segment between the resection margins was fixed in 10% neutral-buffered formalin. Pigs were euthanized under general anesthesia via intravenous potassium chloride. The harvested ureters were serially sectioned at 3 mm intervals, paraffin-embedded, and stained with hematoxylin and eosin. The retrieved stents were gently rinsed with sterile saline, imaged by laser confocal microscope (VK-X3050, Keyence, Japan). Histopathological assessment for the presence and severity of urothelial hyperplasia, inflammation, mucin cell metaplasia/umbrella cell vacuolization, fibrosis, lamina propria edema, and congestion was performed by a pathologist (S.J.S.) according to a previously established classification system. To assess the organisms on ureteral stents, outer, inner, and cross-sectional surfaces were imaged by SEM (IT-500HR, JEOL, Japan). Mineral deposition on retrieved stents was further quantified by measuring Mg and Ca content using ICP-OES (5110, Agilent, USA) after cutting the stents into 5 cm segments.

### 2.12 Radar chart scoring methodology

**Fig. 1:**
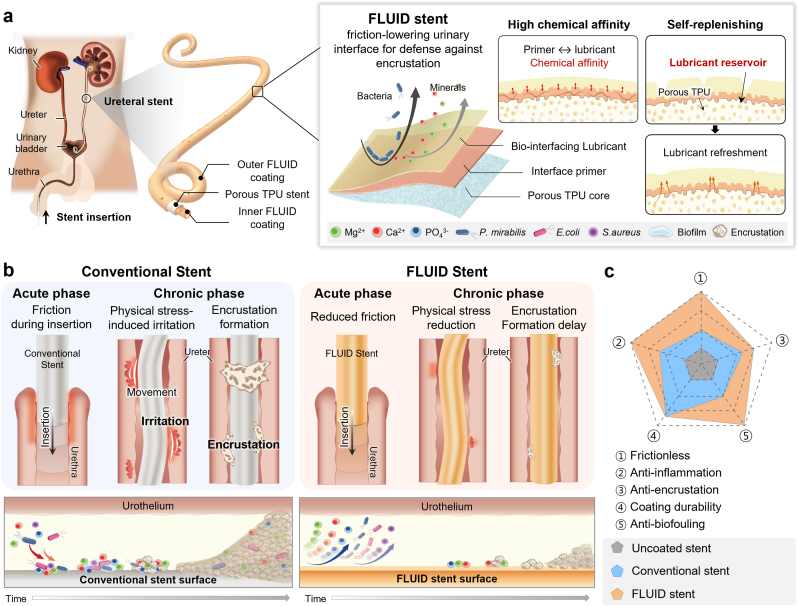
Design and comparative characteristics of the FLUID stent. (a) Structure of the FLUID stent featuring a self-replenishing lubricant layer and a porous TPU core. (b) Comparison of conventional and FLUID stents in acute (insertion) and chronic (long-term indwelling) phases. (c) Comparison of functional characteristics of uncoated, conventional, and FLUIDstents.

The radar chart in Fig. 1c was constructed from experimentally measured values across four key performance dimensions. (i) Frictional behavior was scored as the normalized inverse of the peak pull-out force in the artificial-ureter sliding test described in Section 2.8 (Fig. 3j; lower force corresponds to higher score). (ii) Resistance to encrustation and biofouling was scored as the combined normalized inverse of fluorescence-based protein coverage (albumin and fibrinogen) and bacterial adhesion percentage (*E. coli*, *B. cereus*, *S. aureus*) on the stent surface from the protein and bacterial adhesion tests described in Section 2.9 (Fig. 4b and c), together with the normalized inverse of total Ca and Mg deposition by ICP-OES on retrieved stents at the 4-week time point in the porcine implantation model described in Section 2.11 (Fig. 5g). (iii) Suppression of inflammation was scored as the normalized inverse of the histopathological inflammation score from urothelial tissue analysis at the 4-week time point (Fig. 5d and e). (iv) Coating durability was scored as the surface lubricant retention (relative to the initial fluorescence coverage) after 1000 rubbing cycles in the artificial-ureter friction model described in Section 2.8 (Supplementary Fig. 13). For each dimension, the raw measurements from the FLUID, uncoated TPU, Polaris™ Ultra, Tria™ Soft, and Inlay Optima™ stents were normalized to the best performer in that dimension (set to 1.0), and the resulting scores were plotted as the radar chart axes.

### 2.13 Statistical analysis

Statistical analyses were performed using GraphPad Prism 8 software (GraphPad Software Inc., USA). Unpaired t-tests and one-way analysis of variance (ANOVA) were used for independent *in vitro* experiments, and paired t-tests were applied for comparisons between the left (Tria™) and right (FLUID-coated) ureters within the same animal. For *in vitro* datasets with n = 3, the Shapiro–Wilk test was used to assess normality. When normality was satisfied, parametric t-tests or one-way ANOVA were applied; when normality was not satisfied, non-parametric alternatives (Mann–Whitney *U* test for two-group comparisons, Kruskal–Wallis test for multi-group comparisons) were used. Where parametric and non-parametric tests yielded the same qualitative conclusion, the parametric test result is retained with normality-test confirmation; where the two tests disagreed, the non-parametric result is reported. Data are presented as mean ± s.d. Statistical significance was determined using paired or unpaired t-tests as appropriate (∗*p* < 0.05, ∗∗*p* < 0.01, ∗∗∗*p* < 0.001, ∗∗∗∗*p* < 0.0001, ns, not significant).

## 3 Results and discussion

### 3.1 Design principles of FLUID stent

The FLUID stent integrates a primer-stabilized lubricant layer with a microporous TPU double-J stent body (Fig. 1a). Microporosity extends throughout the stent wall, enabling lubricant retention within the framework and continuous supply to both the luminal and abluminal surfaces. The coating architecture comprises a bio-interfacing lubricant, an interface primer, and a porous TPU core. At the solid interface, the primer stabilizes the lubricant through chemical affinity. Within the stent body, lubricant retained in the porous matrix redistributes to the surface after local depletion during insertion, abrasion, or physiological friction and restores interfacial coverage. This coupling of interfacial anchoring and structural lubricant storage sustains a self-replenishing lubricating layer under repeated mechanical challenge. The stable mobile lubricant interface minimizes direct solid contact and reduces the persistent surface interactions required for adhesion of bacteria and mineral nuclei.

These structural features provide clear advantages during both the acute and chronic phases of ureteral stent implantation (Fig. 1b). During insertion, direct contact between a conventional polymer surface and the urothelium can induce friction-mediated trauma. During prolonged indwelling, the same exposed interface is subjected to repeated physical stress and progressively supports biological deposition, bacterial colonization, and mineral accumulation. By maintaining a stable lubricant interface throughout both phases, FLUID can reduce insertion-related friction, mitigate physical irritation during residence, and delay the early interfacial events that promote biofouling and encrustation. This material architecture can therefore improve lubricity during placement and maintain greater surface stability during long-term indwelling.

The comprehensive performance of ureteral stents can be evaluated across four key domains: frictional behavior, resistance to encrustation and biofouling, suppression of inflammation, and coating durability. These domains represent the principal challenges that compromise the long-term function of conventional stents. The FLUID stent was developed to address these limitations by integrating improvements across all domains through a single multifunctional coating. The radar chart illustrates these multidimensional improvements, showing that the FLUID stent consistently occupies a broader functional profile than both uncoated and conventional stents (Fig. 1c). This comprehensive improvement demonstrates that the anti-adhesive and lubricious surface confer superior performance under physiologically relevant conditions, thereby addressing key shortcomings of current stent technologies.

### 3.2 Fabrication of the FLUID stent

**Fig. 2:**
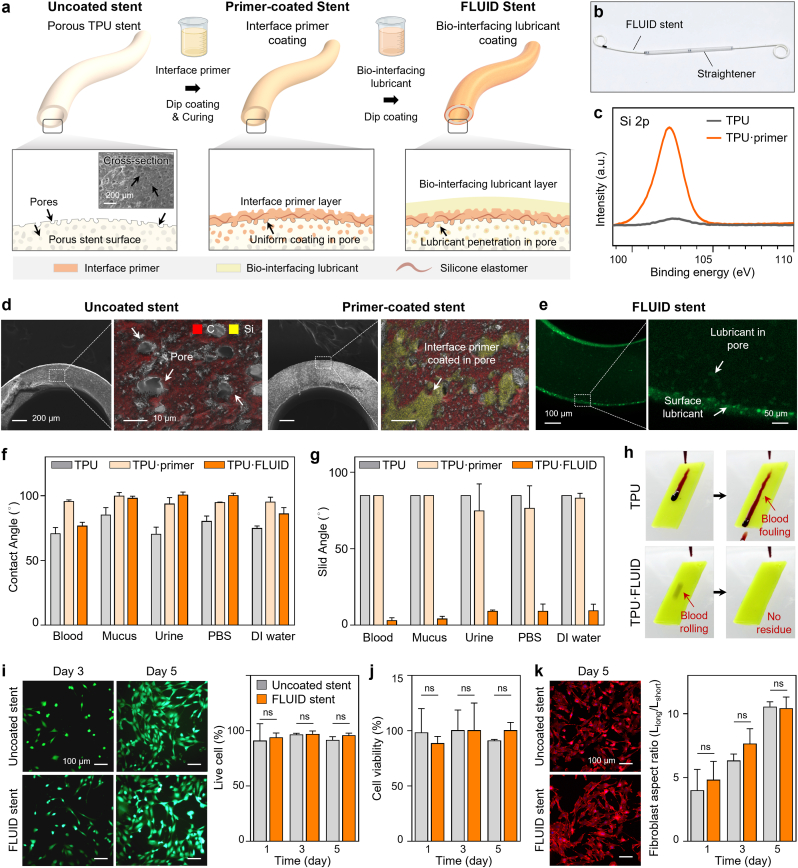
Fabrication and characterization of the FLUID stent. (a) Schematic illustration of the fabrication process of FLUID stents and cross-sectional SEM image of porous TPU stent. (b) Optical image of the FLUID stent. (c) XPS spectra of uncoated and primer-coated stent surfaces. (d) Cross-sectional SEM and EDS images of uncoated and primer-coated stents. (e) Cross-sectional fluorescence microscopic images of FLUID stent surfaces. (f) Contact angle and (g) sliding angle measurements for various biofluids (*n* = 3). (h) Blood adhesion test comparing TPU and TPU-FLUID surfaces. (i) Fluorescence microscopic images of live/dead stained NIH-3T3 fibroblasts, with statistical analysis (*n* = 3). (j) Cell viability evaluation of fibroblasts by CCK-8 assay (*n* = 3). (k) Fluorescence microscopic images of TRITC-phalloidin-stained fibroblasts, with statistical analysis (*n* = 3). Data are shown as mean ± s.d., unpaired *t*-test (∗*p* < 0.05, ∗∗*p* < 0.01, ∗∗∗*p* < 0.001, ∗∗∗∗*p* < 0.0001, ns, not significant).

Building on these design requirements, the FLUID stent was engineered with a multilayered architecture to ensure both coating stability and functional performance. Fig. 2a schematically illustrates the stepwise fabrication of the FLUID stent. The fabrication process involved three sequential steps: (1) extrusion of a porous stent core, (2) application of an interface primer to impart chemical affinity, and (3) coating of a bio-interfacing lubricant that provides low-friction and anti-adhesion properties. First, the stent core was produced by extruding TPU with a foaming agent. The foaming process generated a microporous structure throughout the TPU matrix, effectively increasing the surface area for subsequent coating and facilitating lubricant retention (Supplementary Fig. 1). Porosity analysis indicated an average total porosity of 6.24%, confirming the presence of accessible void volume within the polymer matrix (Supplementary Table 2). Despite this porosity, the tensile strength remained comparable to that of the commercial Tria™ stent, indicating minimal impact on mechanical performance (Supplementary Fig. 2). Second, an interface primer of silicone elastomer was dip-coated onto the porous TPU, uniformly covering the surfaces and pore walls. The methoxy groups (-*Si*-OCH_3_) in the primer are hydrolyzed to generate silanol groups (-*Si*-OH), which primarily self-condense to form a siloxane (Si-O-Si) network. Third, silicone oil was dip-coated onto the primer-treated stent, where its strong affinity for the primer enabled uniform wetting and infiltration of both the surface and porous microstructure. A medical-grade silicone oil was selected as the lubricant, given its established use in biomedical applications and favorable biocompatibility [37,38]. A lubricant with a viscosity of 350 cSt was employed, as preliminary friction tests using oils of 50-10,000 cSt identified it as the most effective for minimizing friction (Supplementary Fig. 3). The silicone oil layer establishes a fluidic interface where surface molecules remain mobile rather than fixed, unlike a solid surface. This fluidic mobility prevents the formation of stable adhesion layers, allowing transient adsorbates to be easily washed away by surrounding fluids. Moreover, the oil layer creates a continuous and uniform lubricating film over the surface, providing a low-friction interface. These steps yielded the final configuration of the FLUID stent (Fig. 2b), consisting of a porous TPU core, an interface primer layer that mediates adhesion, and a lubricant layer that provides durable low-friction and anti-adhesion properties. The fabricated stents were packaged and subsequently sterilized by electron-beam irradiation, enabling immediate clinical use (Supplementary Fig. 4).

**Fig. 3:**
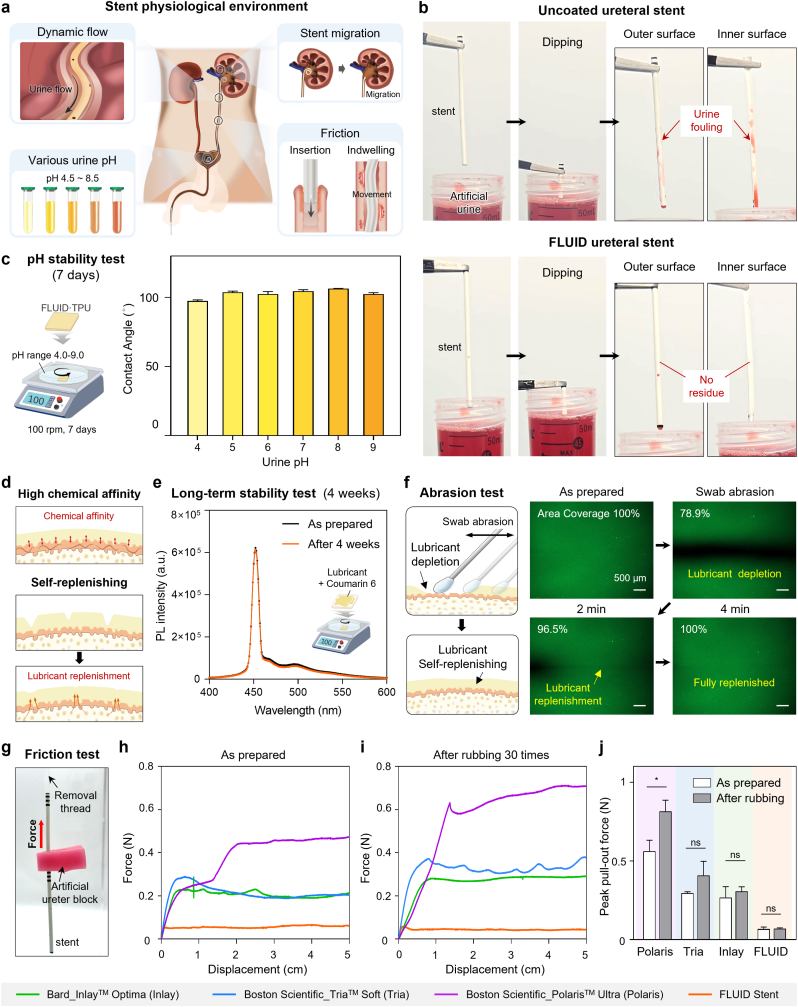
Coating stability of the FLUID stent under physiological environment. (a) Schematic illustration of stent physiological environment. (b) Urine fouling test of uncoated and FLUID stents. (c) Contact angle of FLUID stents after 7 days in artificial urine with different pH values (*n* = 3). (d) Schematic illustration of lubricant retention and refreshment behavior of the FLUID coating. (e) Long-term stability mechanisms and Photoluminescence (PL) intensity of FLUID-coated samples doped with coumarin 6 after orbital shaking at 100 rpm for 4 weeks. (f) Abrasion test and lubricant replenishment of the FLUID coating. (g) Schematic of friction test setup. (h,i) Frictional force-displacement curves of conventional and FLUID stents. (h) as prepared and (i) after 30 rubbings. (j) Peak pull-out force of conventional and FLUID stents before and after rubbing (*n* = 3). Data are shown as mean ± s.d., unpaired *t*-test (∗*p* < 0.05, ∗∗*p* < 0.01, ∗∗∗*p* < 0.001, ∗∗∗∗*p* < 0.0001, ns, not significant).

**Fig. 4:**
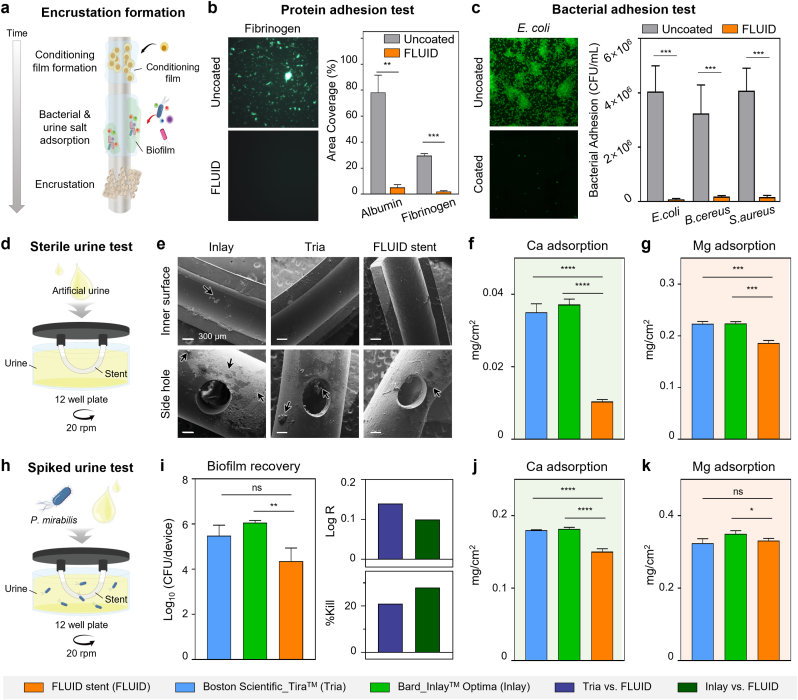
Evaluation of bacterial adhesion and encrustation on FLUID and conventional stents. (a) Schematic illustration of the encrustation process. (b) Protein adhesion test with fluorescence images and quantification of albumin and fibrinogen adsorption on uncoated and FLUID substrates (*n* = 3). (c) Fluorescence images and quantification of *E. coli*, *B. cereus*, and *S. aureus* adhesion on uncoated and coated stents (*n* = 3). (d) Schematic of sterile urine test setup. (e) SEM images of inner surfaces and side holes of FLUID and conventional stents. (f,g) Quantification of Ca and Mg deposition on FLUID and conventional stents under sterile urine conditions (*n* = 3). (h) Schematic of spiked urine test with *P. mirabilis*. (i) Adherent bacterial recovery in spiked urine test (*n* = 3). (j,k) Quantification of Ca and Mg deposition on FLUID and conventional stents under spiked urine conditions (*n* = 3). Data are shown as mean ± s.d., unpaired *t*-test (∗*p* < 0.05, ∗∗*p* < 0.01, ∗∗∗*p* < 0.001, ∗∗∗∗*p* < 0.0001, ns, not significant).

**Fig. 5:**
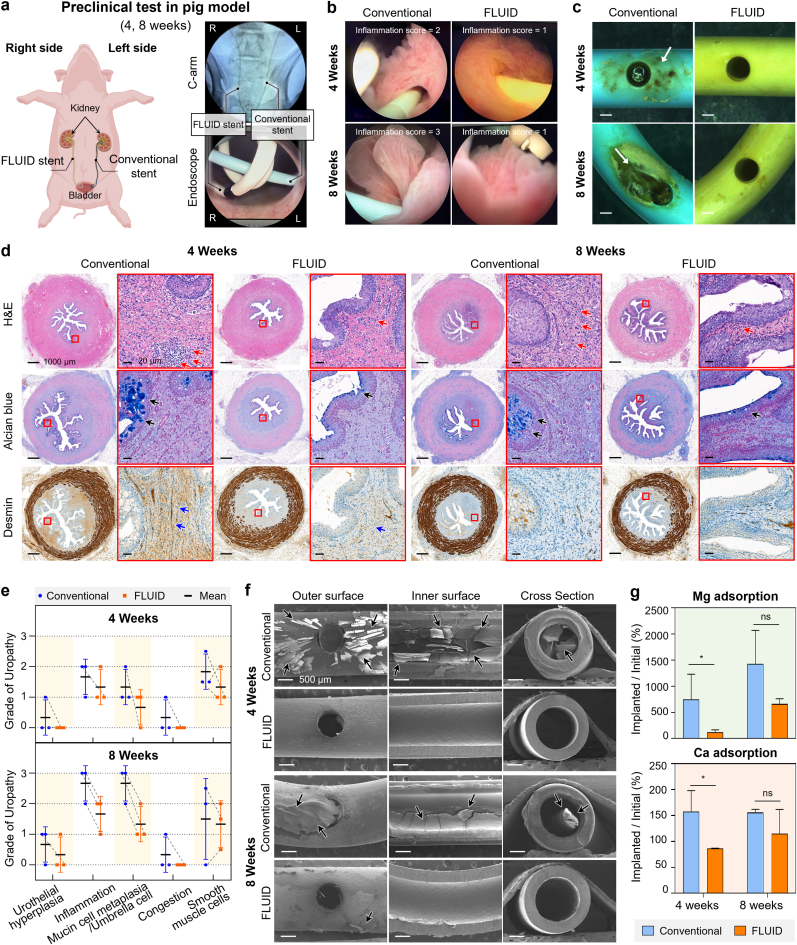
Preclinical evaluation of FLUID stents in a porcine model. (a) Schematic of porcine implantation model with C-arm fluoroscopy and cystoscopy images. (b) Endoscopic images of the ureteral orifice. (c) Optical images of retrieved stents. (d) Histological staining with H&E, Alcian blue, and Desmin. Red arrows indicate inflammation, black arrows indicate mucin cells, and blue arrows indicate smooth muscle cells. (e) Histopathological scoring of urothelial tissues (*n* = 3). (f) SEM images of outer, inner, and cross-sectional surfaces of retrieved stents. Black arrows indicate biomaterial deposition. (g) ICP-OES analysis of Mg and Ca deposition (*n* = 3). Data are shown as mean ± s.d. Statistical significance was determined using paired or unpaired t-tests as appropriate (∗*p* < 0.05, ∗∗*p* < 0.01, ∗∗∗*p* < 0.001, ∗∗∗∗*p* < 0.0001, ns, not significant).

### 3.3 Characterization of FLUID coating

To verify the chemical deposition of the primer and confirm its interaction with the TPU substrate, XPS analysis was subsequently performed (Fig. 2c). The spectra showed a clear increase in the Si2p peak at approximately 101 eV in the primer-coated stent compared with the uncoated TPU stent, indicating the successful introduction of a silicone-based layer, a result further supported by quantitative elemental analysis (Supplementary Table 3), which revealed a higher atomic ratio of Si in the primer-coated sample. Consistent with this surface modification, the friction coefficient of the coated substrate decreased from 1.15 to 0.62, corresponding to an approximately 1.9-fold reduction, indicating that the introduced silicone-based interface effectively lowers interfacial resistance during sliding (Supplementary Fig. 5).

In parallel, structural analysis demonstrated that while distinct micropores were visible throughout the cross-section of the uncoated stent (Fig. 2d), Si signals were detected along the internal pore walls of the primer-coated stent, confirming that the primer solution had penetrated into the porous matrix during dip-coating and extended the coating beyond the external surface. This infiltration is critical, as it ensures that the subsequent lubricant is not confined to the exterior but is also anchored within the internal pore network. To directly visualize this process, confocal fluorescence imaging was performed (Fig. 2e and Supplementary Fig. 6), in which a fluorescently labeled lubricant produced strong signals not only on the external surface but also within the interconnected pores, demonstrating that the lubricant, guided by the primer, effectively infiltrated both the surface and the porous interior. To evaluate the contribution of the porous architecture to lubricant retention, FLUID coatings were applied to stent segments fabricated either with or without a foaming agent during extrusion. The coated samples were then subjected to 30 repeated rubbing cycles against an artificial ureter block, followed by measurement of the frictional force (Supplementary Fig. 7). The stent with pores exhibited only a negligible change in peak pull-out force (approximately 2.2%) before and after coating, whereas the non-porous stent showed a substantial 49.8% increase in friction, indicating that the porous microstructure effectively stabilizes the lubricant layer during mechanical contact. These findings establish that the interface primer is firmly anchored throughout the porous stent and that the lubricant achieves stable retention across both external and internal pore surfaces, thereby providing the layered configuration that underpins the FLUID stent design and ensures long-term stability of the lubricious surface under physiological conditions.

The anti-adhesive performance of the FLUID coating was evaluated by measuring contact and sliding angles at each coating stage in biofluids including blood, mucus, and urine. After application of the primer and FLUID layers, contact angles increased markedly in all tested fluids compared with uncoated TPU (Fig. 2f). For urine in particular, the contact angle increased from 70° for the uncoated stent to 90° for the TPU-primer sample and 100° for the TPU-FLUID sample. These results indicate that the interface primer and FLUID coating substantially altered the intrinsic wettability of the stent surface, which is a prerequisite for anti-adhesive behavior. Because contact angle measurements represent only the static equilibrium of droplets and do not capture their mobility under dynamic conditions, sliding angles were also measured as a more direct indicator of liquid repellency (Fig. 2g). Uncoated TPU and primer-coated samples exhibited high sliding angles in most fluids, reflecting strong droplet pinning and poor de-wetting behavior. In contrast, FLUID-coated samples showed exceptionally low sliding angles, below 15° across all fluids tested, with values as low as 5° in urine and blood. These findings demonstrate that lubricant infusion not only increases static contact angles but also drastically reduces sliding resistance, thereby creating a dynamic surface state that actively prevents liquid adhesion. The practical implications of these wetting properties were further examined through blood adhesion tests (Fig. 2h). On uncoated TPU surfaces, blood spread extensively and left visible residues, consistent with strong fouling. By contrast, on the FLUID-coated surface, deposited blood droplets rolled off completely without leaving detectable traces, even after repeated deposition and removal cycles. These results highlight that the FLUID coating provides robust resistance to fouling by suppressing the adhesion of diverse biofluids.

To ensure clinical applicability, the stability and safety of the FLUID coating were systematically evaluated. The sterilization stability of the coating was first examined by applying the FLUID layer onto a TPU substrate, sealing it within double packaging, and irradiating it with an electron-beam dose of 45 kGy (Supplementary Fig. 8). The contact angle remained over 100° after sterilization, confirming that the coating maintained its physicochemical integrity. Finally, to confirm that these functional improvements were not achieved at the expense of safety, the biocompatibility of the FLUID stent was evaluated through *in vitro* assays.

NIH-3T3 fibroblasts cultured with the stent for up to 5 days showed survival rates above 90% in live/dead staining, comparable to uncoated stents and indicating negligible cytotoxicity (Fig. 2i and Supplementary Fig. 9). Consistent results from CCK-8 assays further demonstrated no significant differences in cell viability between groups (Fig. 2j). Morphological analysis with tetramethylrhodamine isothiocyanate (TRITC)-phalloidin staining revealed that fibroblasts maintained normal shape and intact actin filaments without signs of rounding, cytoskeletal disruption, or loss of stress fibers (Fig. 2k and Supplementary Fig. 10). Quantitative measures of aspect ratio and actin fluorescence intensity also matched controls. These findings establish that the FLUID stent does not provoke cytotoxic or inflammatory responses, consistent with the preliminary biocompatibility of the FLUID stent. Notably, additional biocompatibility tests were conducted in accordance with ISO 10993 standards for medical devices, including cytotoxicity, pyrogenicity, intracutaneous reactivity, and acute systemic toxicity. All results met the acceptance criteria (Supplementary Table 4), supporting the preliminary biocompatibility of the FLUID stent for potential medical use.

### 3.4 Stability of the FLUID stent in physiological environments

During placement, ureteral stents are exposed to continuously changing physiological environments, including variations in urinary pH, constant mechanical motion, and prolonged immersion in biofluids. To ensure clinical reliability, the coating must retain its structural integrity and lubricious properties under these diverse stress conditions. Accordingly, the long-term stability of the FLUID coating was evaluated in terms of chemical resistance under urine-relevant pH conditions, lubricant retention under continuous hydrodynamic agitation, resistance to repetitive mechanical damage, and preservation of surface coverage and morphology. Fig. 3a schematically illustrates the major stress factors encountered by ureteral stents during placement, including pH fluctuations [39,40], hydrodynamic flow [20,41], and repetitive mechanical contact. After implantation, the device is continuously exposed to urine, where variations in composition, ionic strength, and flow dynamics can influence surface stability. Although urinary pH is typically between 5.5 and 7.0, pathological conditions can cause substantial deviations. Urease-producing infections may increase the pH above 8, whereas metabolic acidosis can decrease it below 4.5. In addition to these chemical stresses, hydrodynamic shear from continuous urine flow and patient movement subjects the coating to persistent mechanical perturbation. Such combined chemical and physical challenges can degrade conventional coatings and compromise their lubricity over time. To evaluate whether the FLUID stent maintains long-term stability under these conditions, systematic tests were performed to assess its chemical stability across pH extremes, lubricant retention under hydrodynamic flow, and frictional performance after repeated mechanical contact.

The urine fouling test evaluated the susceptibility of stents to early surface contamination (Fig. 3b and Supplementary Movie 1). The uncoated and FLUID stents were immersed in red-dyed simulated urine. The uncoated stent exhibited distinct deposits on both luminal and abluminal surfaces, confirming its susceptibility to rapid fouling. In contrast, the FLUID-coated stent remained free of staining, indicating that the lubricious layer was uniformly applied and immediately effective in preventing urine-induced contamination. To further examine coating stability, FLUID-coated samples were incubated for 7 days in artificial urine adjusted to pH values between 4.0 and 9.0 (Fig. 3c). Across this range, water contact angles were consistently maintained between 97.4 ± 0.8° and 106.3 ± 0.2°, with no signs of surface degradation or loss of lubricity. These findings demonstrate that the FLUID coating preserves its hydrophobic and lubricious properties under both normal urinary pH (5.5-7.0) and pathological extremes such as infection-driven alkalinization or metabolic acidosis, underscoring its stability during long-term placement.

Following implantation, ureteral stents are continuously exposed to urine flow; therefore, maintaining long-term stability of coating is critical for reliable performance. The FLUID coating achieved excellent durability owing to the strong chemical affinity between the primer and lubricant layers, further reinforced by the capillary-driven retention of the lubricant within the microporous TPU matrix (Fig. 3d) [28,42]. This porous architecture acts as a robust physical framework that shields the lubricant from high hydrodynamic shear and mechanical friction, preventing premature depletion. To assess lubricant retention, silicone oil doped with coumarin 6 was used as a fluorescent tracer and immersed in DI water under vigorous orbital shaking at 100 rpm for 4 weeks (Fig. 3e). Photoluminescence intensity remained essentially unchanged after 4 weeks of continuous hydrodynamic agitation, demonstrating that the lubricant layer was stably preserved even under sustained and aggressive flow conditions. To evaluate lubricant retention under dynamic conditions, the stent was inserted into an artificial ureter and immersed in artificial urine, followed by 1000 cycles of shaking (Supplementary Fig. 11). The lubricant was labeled with coumarin 6 to quantify both the fraction released into the urine and the fraction remaining on the stent surface. Fluorescence intensity measured from the surrounding urine indicated that up to 250 cycles, loosely associated lubricant was partially removed from the surface. At 500 and 1000 cycles, the fluorescence signal reached a plateau, indicating stabilized lubricant loss. Surface fluorescence coverage analysis further confirmed that, although statistically significant changes were observed, more than 97% of the initial coverage was retained even after 1000 cycles. These observations suggest that loosely attached lubricant is removed during the initial washing process, while the remaining lubricant persists on the surface and maintains a stable coating layer under dynamic conditions.

The self-replenishing behavior of the FLUID coating under localized mechanical damage was evaluated using a swab abrasion test (Fig. 3f). Following swab-induced abrasion, partial lubricant depletion was intentionally introduced, resulting in a reduced surface area coverage of 78.9%. Despite this localized loss, fluorescence imaging revealed rapid recovery of the lubricant layer, reaching complete surface coverage within 4 min. This recovery is primarily driven by lubricant released from the microporous TPU matrix [43], while the depleted region is refilled as surrounding lubricated areas relax toward the damaged site. These fluorescence and surface-coverage analyses further verify that the coating morphology and interfacial lubricant layer remain stable even after localized damage. Even when local depletion occurs, the lubricant layer is rapidly restored, preventing prolonged exposure of the underlying porous substrate. This dynamic recovery mechanism is critical for maintaining low-friction conditions and for suppressing secondary biological events such as bacterial adhesion and mineral nucleation during long-term indwelling. Given that the coating was designed to impart high lubricity, it was necessary to evaluate whether this property might inadvertently reduce anchoring strength and promote stent migration.

### 3.5 Mechanical reliability and sustained low-friction performance

Given that the coating was designed to impart high lubricity, it was necessary to evaluate whether this property might inadvertently reduce anchoring strength and promote stent migration. To address this, anchoring performance was assessed using a pull-out test in an artificial ureter model (Supplementary Fig. 12). Both uncoated and FLUID-coated stents exhibited comparable peak forces (230 ± 20 mN vs. 210 ± 10 mN), with no statistically significant difference. These results suggest that stent migration is primarily governed by the disengagement behavior of the pigtail curl and is not substantially influenced by the FLUID coating. Therefore, the FLUID stent enhances surface lubricity without inducing mechanical dislodgement, ensuring stable positioning during implantation.

Frictional properties of the FLUID stent were compared with those of conventional hydrophilic and hydrophobic stents (Fig. 3g and h). The hydrophilic stent tested was the Polaris™ Ultra (Polaris), whereas the hydrophobic group included the Tria™ Soft (Tria) and Inlay Optima™ (Inlay). To simulate insertion, stent shaft segments were attached to a thread and drawn through an artificial ureter block. In accordance with surgical practice, all conventional stents, with the exception of the FLUID stent, were soaked in PBS for 10 min prior to testing in order to maximize lubricity. Because this setup was designed to mimic clinical insertion through a confined ureter-like channel, friction was compared using pull-out force as a clinically relevant metric rather than a classical coefficient of friction obtained from a flat tribological configuration. The FLUID stent demonstrated a peak pull-out force of 0.07 ± 0.02 N, which was approximately 7.9-fold lower than the hydrophilic Polaris stent (0.56 ± 0.07 N) and about 4-fold lower than the hydrophobic Tria (0.29 ± 0.01 N) and Inlay (0.27 ± 0.07 N). This pronounced reduction indicates that the FLUID stent achieves a level of lubricity surpassing both hydrophilic and hydrophobic conventional stents, despite requiring no pre-soaking step. Frictional stability was further assessed under repeated contact by rubbing stent sections against the artificial ureter block 30 times prior to measurement (Fig. 3i and j). The hydrophilic Polaris stent showed a significant increase in friction, rising to 0.82 ± 0.070 N, which was attributed to surface coating layer damage under repetitive stress. In contrast, the FLUID stent maintained a nearly unchanged frictional force (0.07 ± 0.004 N) even after 30 repeated rubbing cycles, demonstrating superior wear resistance compared to conventional hydrophilic coatings which showed functional degradation (0.82 ± 0.070 N) due to surface delamination. Lubricant retention on the stent surface was further evaluated after 1000 cycles of physical friction in an artificial ureter model (Supplementary Fig. 13). Fluorescence imaging confirmed that the lubricant layer remained preserved, with high surface coverage maintained despite repeated mechanical abrasion. This sustained lubricity proves that the lubricant is not merely a transient layer but is stably anchored and replenished by the porous framework, effectively withstanding the repetitive mechanical stresses encountered during ureteral peristalsis.

### 3.6 Functional evaluation of bacterial adhesion and encrustation on FLUID

Clinically, encrustation is one of the most common complications associated with long-term ureteral stent placement, in which mineral deposits accumulate on the stent surface, obstructing urine flow and making removal difficult. This process follows a well-characterized, causally linked cascade that is initiated at the implanted surface. Within minutes of placement, urinary proteins (notably Tamm–Horsfall protein, albumin, and immunoglobulins) adsorb onto the stent surface and assemble a conditioning film [44,45] that alters local surface chemistry and exposes binding sites for bacterial adhesins. This protein-conditioned interface subsequently accelerates bacterial adhesion and biofilm maturation, and the resulting biofilm matrix together with local urine alkalinization by urease-producing organisms such as *P. mirabilis* promotes supersaturation of Ca^2+^, Mg^2+^, and PO_4_^3−^ and favors heterogeneous nucleation of calcium phosphate, calcium oxalate, and struvite on the already-fouled surface. Because each downstream step requires a stable, molecularly anchored interface to initiate, disruption of the earliest conditioning-film step is mechanistically sufficient to attenuate the downstream stages [17,46,47]. As illustrated in Fig. 4a, encrustation begins as urinary components adsorb onto the stent surface after implantation. Initially, urinary proteins adhere to the surface, forming a conditioning film that facilitates bacterial attachment and colonization [16,48]. Subsequently, bacterial proliferation leads to the development of a biofilm, within which the deposition and crystallization of inorganic ions are accelerated, resulting in progressive encrustation. Specifically, urinary proteins such as Tamm–Horsfall protein, albumin, and immunoglobulins adsorb onto the implanted surface within minutes of placement to assemble a protein-conditioned interface that exposes binding sites for bacterial adhesins and accelerates downstream biofilm maturation. The resulting biofilm matrix together with local urine alkalinization by urease-producing organisms such as *P. mirabilis* promotes supersaturation of Ca^2+^, Mg^2+^, and PO_4_^3−^ and favors heterogeneous nucleation of calcium phosphate, calcium oxalate, and struvite on the already-fouled surface. Such deposits can gradually develop even in sterile urine through supersaturation of urinary constituents; however, the process is markedly accelerated in the presence of infection, as urease-producing bacteria raise the urinary pH and promote mineral precipitation [40]. The FLUID coating was designed to systematically suppress this cascade of encrustation. Its fluidic surface simultaneously inhibits the formation of the conditioning film caused by initial protein adsorption and the subsequent biofilm formation induced by bacterial adhesion, thereby mitigating mineral deposition and encrustation progression. Because encrustation varies widely among individuals depending on urinary composition, infection status, and placement duration, and is therefore difficult to predict, such a multistage inhibitory mechanism provides an essential preventive strategy for clinical use [39].

### 3.7 Inhibition of protein and bacterial adhesion

Protein adsorption, a critical early step in biofouling, was evaluated using albumin and fibrinogen as representative plasma-derived proteins (Fig. 4b and Supplementary Fig. 14). Albumin, which commonly appears in urine during renal dysfunction [49,50], and fibrinogen, which can enter the urinary tract during hematuria or inflammation [51,52], were selected as model proteins. Fluorescence microscopy revealed extensive surface coverage on uncoated stents, whereas FLUID-coated stents exhibited only minimal fluorescence, indicating markedly reduced adsorption. Quantitative analysis of fluorescence intensity further confirmed this observation, with both albumin and fibrinogen showing significantly lower adhesion on FLUID-coated samples than on uncoated stents. These results demonstrate that the lubricious anti-adhesive layer of the FLUID stent effectively suppresses the initial adsorption of urinary proteins, thereby delaying conditioning film formation. To further assess whether the FLUID coating can mitigate bacterial colonization, *in vitro* adhesion assays were performed using three representative uropathogens: *E. coli*, the most common cause of urinary tract infections [53,54], and two Gram-positive species with strong biofilm-forming ability, *B. cereus* and *S. aureus* (Fig. 4c and Supplementary Fig. 15) [44,55]. Uncoated and FLUID-coated samples were incubated for 72 h, after which bacterial attachment was evaluated by fluorescence microscopy. Extensive colonization was observed on uncoated surfaces, whereas FLUID-coated surfaces exhibited minimal bacterial adhesion. Quantitative analysis confirmed that adhesion was reduced by more than 94% across all tested strains, demonstrating the potent and broad-spectrum anti-adhesive performance of the FLUID coating. These findings verify that the FLUID coating effectively interrupts the sequential initiation steps of encrustation, including conditioning film formation and bacterial adhesion, thereby preventing the cascade that ultimately leads to biofilm development and mineral deposition.

### 3.8 *In vitro* assessment of mineral deposition

Following the suppression of protein and bacterial adhesion, the next step of the encrustation cascade, mineral deposition, was investigated using *in vitro* models under both sterile and infection-mimicking conditions. To simulate mineral deposition under sterile conditions, an *in vitro* encrustation model using artificial urine was employed (Fig. 4d) [56]. Stents were incubated under continuous rotation at 20 rpm for 2 weeks. Macroscopic deposits became visible on all stents, and SEM analysis revealed mineral accumulation, particularly around external side holes rather than within the lumen (Fig. 4e and Supplementary Fig. 16). Urinary encrustation is primarily composed of mineral crystals such as calcium oxalate (CaC_2_O_4_), calcium phosphate (Ca_10_(PO_4_)_6_(OH)_2_), and magnesium ammonium phosphate (NH_4_MgPO_4_·6H_2_O). Therefore, deposition of the principal elements Ca and Mg was quantitatively analyzed to evaluate encrustation formation. Quantitative assays of Ca and Mg deposition confirmed that the FLUID stent reduced Ca deposition by 70% and Mg deposition by about 17% compared with conventional stents (Fig. 4f and g). These findings suggest that the coating primarily suppresses Ca deposition, the principal driver of encrustation under sterile conditions, while also reducing Mg accumulation to a measurable extent. To evaluate infection-associated encrustation, artificial urine was further spiked with *P. mirabilis* (Fig. 4h). After 2 weeks of incubation, optical and SEM analyses revealed more extensive deposits than under sterile conditions (Supplementary Fig. 17). To assess biofilm formation, adherent bacteria were recovered from the stent surface and quantified (Fig. 4i). Biofilm-associated bacteria are notoriously resistant to antibiotics and immune clearance, often resulting in chronic infection that necessitates device removal. Log-transformed counts showed significantly fewer adherent bacteria on FLUID stents compared with conventional stents, with statistically significant reductions relative to Inlay. Anti-biofilm performance was further expressed using Log reduction (Log R) and percentage reduction (%Reduction), calculated as:

(1) $$\mathrm{Log}\;R={\mathrm{Log}}_{10}\left(N_{\text{conventional}}/N_{\text{FLUID}}\right)$$

(2) $$\%Reduction=\left(1-\frac{N_{\text{FLUID}}}{N_{\text{conventional}}}\right)\times 100$$

N_conventional_ is the number of bacteria recovered from conventional stents, and N_FLUID_ is the number recovered from FLUID stents. Log R quantifies the fold reduction in bacterial counts relative to the control, whereas %*Reduction* expresses this reduction as a percentage, providing an intuitive measure of the inhibitory capacity of the FLUID coating. Based on these analyses, the FLUID stent achieved approximately 21% reduction (Log R ≈ 0.10) compared with Tria and 28% reduction (Log R ≈ 0.14) compared with Inlay. We note that these values represent modest reductions in mature biofilm-associated bacterial recovery and are distinct from the >94% reduction in planktonic bacterial adhesion observed in the 72 h direct adhesion assay against *E. coli*, *B. cereus*, and *S. aureus*. This difference reflects the mechanism of action of the FLUID coating: the mobile lubricant interface is designed as an anti-adhesion surface that suppresses conditioning-film formation and early bacterial attachment but does not disrupt mature biofilm once it has formed. *P. mirabilis* is additionally a particularly aggressive biofilm-forming uropathogen that produces urease, alkalinizes local urine, and generates crystalline biofilms that anchor within mineral deposits, such that 14-day *P. mirabilis* biofilms represent a stringent benchmark that exceeds the anti-adhesion scope of the FLUID coating. These results indicate that FLUID is most effective at the early, anti-adhesion stage of the encrustation cascade and that combination strategies with antimicrobial surface chemistries may be needed for superior performance against mature infection-associated biofilm.

### 3.9 Preclinical *in vivo* evaluation of FLUID stents in a porcine model

To evaluate the *in vivo* performance of the FLUID stent, 6 Fr, 20 cm ureteral stents were implanted into a porcine model, whose urinary tract anatomy and size closely resemble those of humans (Fig. 5a) [57,58]. In clinical practice, ureteral stents are generally retained for a short duration (days to weeks) in cases of acute infection or after surgery, whereas prolonged indwelling (several months) is required for chronic conditions such as ureteral stricture, renal insufficiency, or ureteral obstruction [17,59]. Based on these clinical indications, implantation periods of 4 weeks and 8 weeks were selected for short-term and long-term evaluation, with three animals assigned to each duration. Sample size was based on ethical considerations and institutional guidelines for large-animal experimentation, consistent with exploratory preclinical studies. All assessments, including endoscopic, histological, and surface analyses, were graded on a four-point scale: none (0), mild (1), moderate (2), or severe (3). The Boston Scientific Tria™ stent, which had demonstrated favorable *in vitro* performance in friction reduction, biofouling resistance, and ion deposition, was selected as the control. The study design enabled side-by-side comparison within the same animal, with one ureter receiving the FLUID stent and the contralateral ureter receiving the Tria stent, thereby minimizing inter-animal variability and ensuring consistent physiological conditions for evaluation.

### 3.10 Endoscopic evaluation and surface assessment after implantation

Endoscopic evaluation provided direct visualization of tissue responses at the ureteral orifice (Fig. 5b). At 4 weeks, conventional stents induced pronounced edema and erythema with an average inflammation score of 2, whereas the FLUID stent showed only mild mucosal changes with a score of 1. By 8 weeks, inflammation further increased around the conventional stent (score 3), while the FLUID stent consistently remained at 1, indicating sustained biocompatibility. These findings suggest that the lubricious coating of the FLUID stent minimizes mucosal irritation and friction-induced trauma, thereby interrupting the early inflammatory cascade. This *in vivo* behavior was consistent with the *in vitro* observations of reduced surface fouling and frictional forces, confirming the coating's biological compatibility. Gross examination of retrieved stents further highlighted clear differences between groups (Fig. 5c). After 4 and 8 weeks of indwelling, conventional stents exhibited extensive macroscopic fouling and greenish deposits, particularly around side holes where urinary components tend to accumulate. In contrast, the FLUID stent maintained a macroscopically clean surface, with side holes remaining fully patent. These findings indicate that the lubricant-anchored coating effectively resists organic and crystalline fouling under physiological flow, preserving luminal patency during implantation.

### 3.11 Histological evaluation of ureteral tissue

Histological analysis was performed to evaluate local tissue responses and long-term biocompatibility. Ureteral samples were stained with H&E, Alcian Blue, and Desmin, and blinded pathological scoring was conducted across parameters including urothelial hyperplasia, inflammation, mucin cell metaplasia or umbrella cell alterations, vascular congestion, smooth muscle integrity, fibrosis, and lamina propria edema (Fig. 5d and e and Supplementary Table 5). Alcian Blue staining highlighted mucin-producing cells, whereas Desmin staining delineated smooth muscle organization, enabling comprehensive assessment of epithelial and stromal remodeling. After 4 weeks, the FLUID group tended to exhibit lower histological scores than conventional stents, characterized by reduced inflammation and minimal epithelial or muscular alterations. These differences became more apparent at 8 weeks, as the FLUID stent preserved organized smooth muscle layers and limited mucin cell metaplasia, whereas conventional stents showed progressive epithelial thickening and stromal fibrosis. Paired analysis revealed that most histopathological parameters were consistently comparable or lower with FLUID stents, suggesting that the lubricant-anchored coating may attenuate chronic inflammation and slow the cascade of tissue remodeling associated with long-term stent implantation.

### 3.12 Surface fouling and mineral deposition analysis

Since the encrustation and biofilm fouling are key factors that compromise long-term stent performance, retrieved devices were further examined by SEM and ICP-OES to assess surface deposition and mineral accumulation (Fig. 5f and g). All implantations were performed under sterile conditions, and no distinct biofilm layers were detected in either group, consistent with the aseptic environment. SEM analysis revealed that conventional stents showed noticeable organic residue on both the luminal and outer surfaces, particularly around side holes, whereas FLUID stents exhibited minimal surface films with no visible occlusion (Fig. 5f). Encrustation typically associated with Ca or Mg precipitation was rarely observed in either group through EDS imaging. Nonetheless, elemental quantification by ICP-OES demonstrated clear compositional differences (Fig. 5g). After both 4 and 8 weeks, Ca and Mg deposition were consistently lower on FLUID stents than on conventional controls. Quantitatively, Mg accumulation was reduced by approximately 84.1% after 4 weeks and 54% after 8 weeks, while Ca deposition decreased by 45.1% and 26%, respectively. The reduction was statistically significant at 4 weeks, indicating that the coating effectively suppresses early-stage mineral deposition. Although the difference was observed to be insignificant at 8 weeks, FLUID stents maintained lower ionic accumulation, supporting the functional stability of their anti-encrustation performance.

### 3.13 Limitations of the current *in vivo* validation

Our *in vivo* validation has several limitations. First, although the coating system met ISO 10993 biocompatibility standards, these tests do not establish long-term safety and should be interpreted as preliminary evidence rather than definitive validation. Dedicated biological safety evaluation of the final finished device, including sensitization, genotoxicity, and sub-chronic or chronic systemic toxicity testing, will be required before clinical translation. Second, the contralateral within-subject design cannot fully exclude potential cross-contamination between the FLUID and control sides. This design was selected to minimize inter-animal variability in urine composition, renal physiology, and systemic factors; however, future studies should incorporate independent-group designs and dedicated analyses of lubricant distribution and cross-transfer. Third, *in vivo* lubricant behavior, including emulsification, migration, chemical degradation, biodistribution, and elimination, was not directly quantified. Although the high hydrophobicity and water immiscibility of medical-grade silicone oil, the dynamic-flow lubricant retention assay, and the absence of overt kidney or bladder injury in 8-week histology suggest limited systemic exposure, direct quantitative analyses are required. Future studies should therefore include GC-MS quantification of silicone oil in urine, ICP-MS quantification of silicon in renal, bladder, and distant tissues, and systemic biochemistry panels including BUN, creatinine, ALT, AST, and CBC in larger cohorts. Fourth, the porcine implantation experiments were conducted under sterile conditions, which do not fully reflect clinically relevant infection-associated scenarios. We intentionally employed a hybrid validation strategy, combining porcine implantation studies for host-device interactions with a controlled *P. mirabilis* model for infection-associated biofouling and encrustation. Dedicated *in vivo* infection-challenge studies under clinically relevant conditions remain an important direction for future investigation. Longer-term implantation studies, including extended observation beyond 12 weeks and dedicated *in vivo* infection models, such as a rabbit intravesical stent-curl surrogate and/or an antibiotic-modulated large-animal protocol, will be necessary to establish long-term safety, lubricant stability, and anti-encrustation performance.

Despite these limitations, the porcine model was selected because of its close anatomical and physiological similarity to the human urinary tract, which is important for translational evaluation of ureteral stents. Within the scope of this mid-term proof-of-concept study, the FLUID stent reduced mechanical irritation, inflammation, biofouling, and mineral deposition compared with conventional double-J stents. These findings provide preliminary preclinical evidence that FLUID may serve as a clinically relevant strategy for improving the safety and durability of long-term ureteral stents, pending further validation in larger, longer-term, and infection-inclusive studies.

## 4 Conclusions

In this work, we developed the FLUID stent as a SLIPS-based ureteral stent platform that integrates a silicone elastomer primer, a medical-grade silicone lubricant, and a microporous TPU scaffold to form a wall-distributed lubricant reservoir. This architecture provides a stable lubricating interface under dynamic urinary-mimicking conditions and supports lubricant retention and replenishment from the internal pore network after localized depletion. Compared with representative commercial double-J stents, FLUID reduced insertional friction by 7.9-fold without a pre-hydration step and maintained low friction after repeated mechanical stress.

Beyond friction reduction, the FLUID coating attenuated several early events associated with the encrustation cascade. *In vitro* assays showed reduced protein adsorption, reduced planktonic bacterial adhesion, and decreased mineral deposition on FLUID surfaces. Under sterile artificial urine conditions, FLUID reduced Ca and Mg accumulation compared with conventional stents. In the *P. mirabilis*-spiked infection model, FLUID showed a modest reduction in mature biofilm-associated bacterial recovery, consistent with its role as an anti-adhesion interface rather than a bactericidal coating. These findings indicate that FLUID primarily acts by limiting surface conditioning and early attachment events that precede biofilm maturation and heterogeneous mineral nucleation.

Preclinical porcine implantation further supported the functional relevance of this coating strategy *in vivo*. Over the 8-week study period, FLUID stents maintained lumen patency, reduced urothelial inflammation, and showed lower mineral accumulation than conventional controls. Histological and elemental analyses provided preliminary evidence of tissue compatibility and reduced early-stage encrustation under sterile implantation conditions. However, the present study should be interpreted as a proof-of-concept preclinical validation rather than definitive evidence of long-term clinical performance.

Importantly, FLUID is PFAS-free and antibiotic-free, and is fabricated from clinically established polymeric materials using a scalable coating process compatible with full-length double-J stents. PDMS-based silicone oils are not classified as persistent organic pollutants and have been reported to undergo environmental degradation without substantial aquatic bioaccumulation, suggesting a potentially favorable environmental profile relative to fluorinated lubricants commonly used in SLIPS. Nevertheless, comprehensive life-cycle assessment, long-term silicone biodistribution, and lubricant elimination analyses were beyond the scope of this study.

Overall, the FLUID stent provides a clinically relevant proof-of-concept platform for reducing friction, early biofouling, and mineral deposition in ureteral stents. Further preclinical studies with larger cohorts, extended implantation periods beyond 12 weeks, dedicated infection models, renal function monitoring, and quantitative lubricant biodistribution analyses will be required to establish its long-term safety, durability, and clinical benefit.

## CRediT authorship contribution statement

**Yejin Jo:** Conceptualization, Investigation, Methodology, Visualization, Writing – original draft, Writing – review & editing. **Yeontaek Lee:** Conceptualization, Investigation, Writing – original draft, Writing – review & editing. **Sungun Bang:** Investigation, Methodology. **Kayoung Son:** Investigation, Methodology. **Kijun Park:** Conceptualization, Investigation, Writing – review & editing. **Dokyun Kim:** Methodology. **Soo A Kim:** Investigation. **Seonghyeon Eom:** Methodology. **Inhee Choi:** Investigation. **Su-Jin Shin:** Methodology, Visualization. **Kyo Chul Koo:** Conceptualization, Investigation, Methodology, Supervision, Writing – original draft, Writing – review & editing. **Jungmok Seo:** Conceptualization, Funding acquisition, Methodology, Supervision, Writing – original draft, Writing – review & editing.

## Declaration of competing interest

The authors declare the following financial interests/personal relationships which may be considered as potential competing interests: Y.L. (chief executive officer) and J.S. (chief executive officer) are affiliated with Lynk Solutec. If there are other authors, they declare that they have no known competing financial interests or personal relationships that could have appeared to influence the work reported in this paper.

## Data Availability

Data will be made available on request.
