# Supplementary material for: Lubricious anti-adhesive interface prevents friction, biofilm, and encrustation in long-term indwelling ureteral stents
Source: Mater Today Bio. 2026 Jun 29;39:103405. doi: 10.1016/j.mtbio.2026.103405 (PMC13355568; doi:10.1016/j.mtbio.2026.103405)
Supplement: Multimedia component 1 [file mmc1.docx]

Supplementary Information

**Lubricious Anti-Adhesive Interface Prevents Friction, Biofilm, and Encrustation in Long-Term Indwelling Ureteral Stents**

Yejin Jo^1^, Yeontaek Lee^1^, Sungun Bang^2^, Kayoung Son^1^, Kijun Park^3^, Dokyun Kim^4^, Seonghyeon Eom^5^, Inhee Choi^5,6^, Su-Jin Shin^7^, Kyo Chul Koo^2,*^, Jungmok Seo^1,*^

Yejin Jo and Yeontaek Lee contributed equally to this work.

*Jungmok Seo. E-mail: [jungmok.seo@yonsei.ac.kr](mailto:jungmok.seo@yonsei.ac.kr)

*Kyo Chul Koo. Email: [gckoo@yuhs.ac](mailto:gckoo@yuhs.ac)


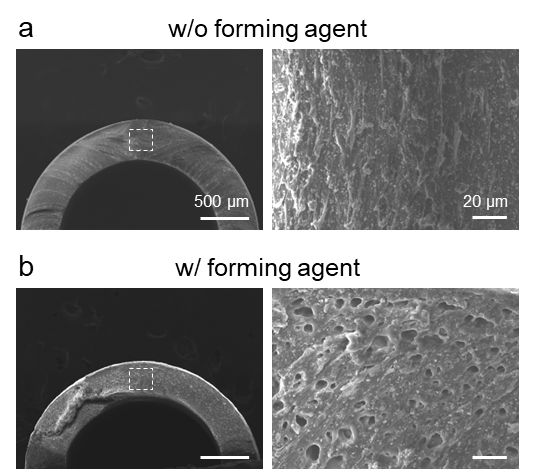


Supplementary Fig. 1

Cross-sectional scanning electron microscopy (SEM) images of stent materials. **a** Sample fabricated without foaming agent, showing a dense internal morphology. **b** Sample fabricated with foaming agent, exhibiting porous microstructure.


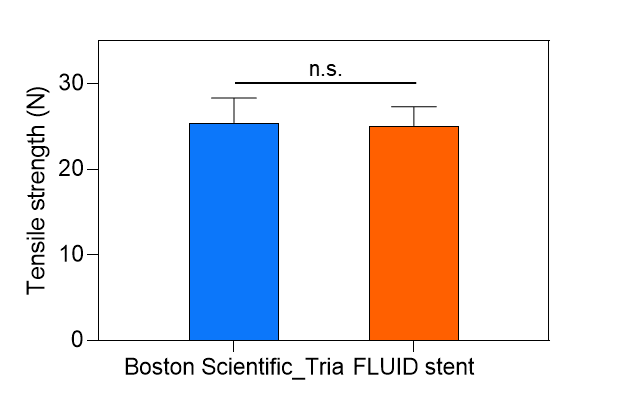


Supplementary Fig. 2

The tensile strengths of Boston Scientific_Tria and FLUID stent (*n* = 2). The microporous structure does not significantly reduce the tensile strength compared to commercial Tria^TM^ stent. Data are shown as mean ± s.d., unpaired t-test (**p* < 0.05, ***p* < 0.01, ****p* < 0.001, *****p* < 0.0001, ns, not significant).


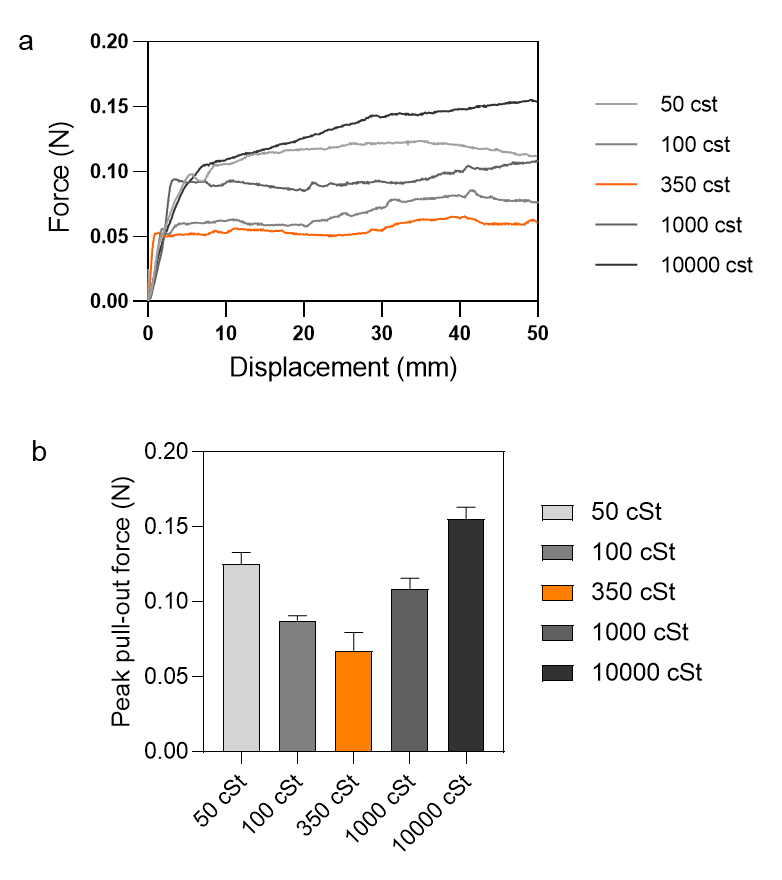


Supplementary Fig. 3

**a** Frictional force–displacement curves and **b** Peak pull-out force of FLUID stents coated with silicone oils of different viscosities (50, 100, 350, 1000, and 10000 cSt). (*n* = 3) Data are shown as mean ± s.d., unpaired t-test (**p* < 0.05, ***p* < 0.01, ****p* < 0.001, *****p* < 0.0001, ns, not significant).

**
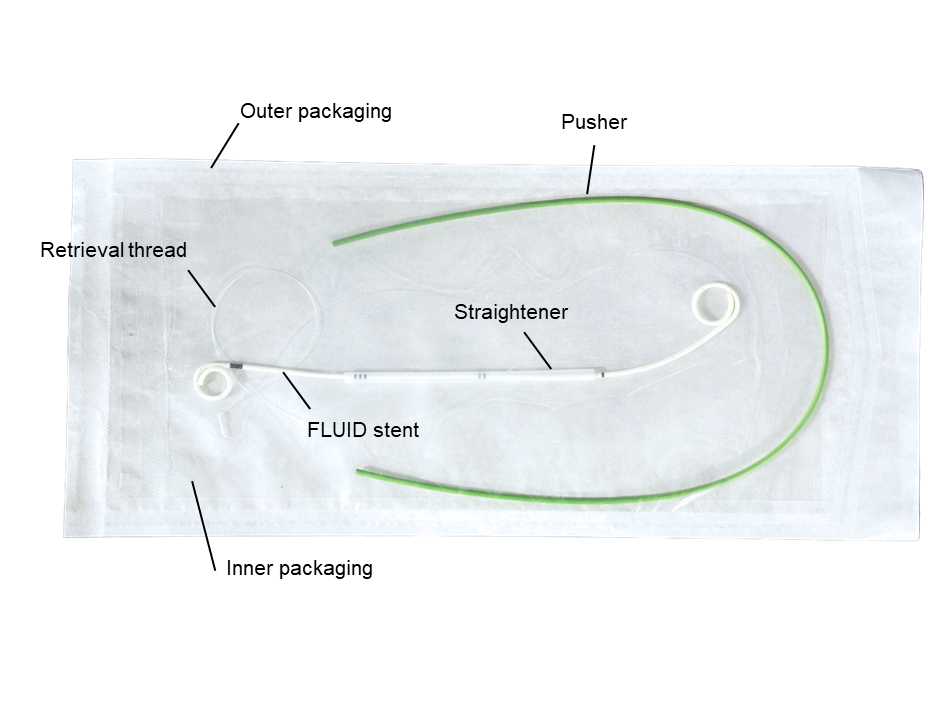
**

Supplementary Fig. 4

Final configuration of the FLUID stent with an integrated pusher and straightener, sterilized by electron-beam irradiation and packaged in a clinically applicable form.


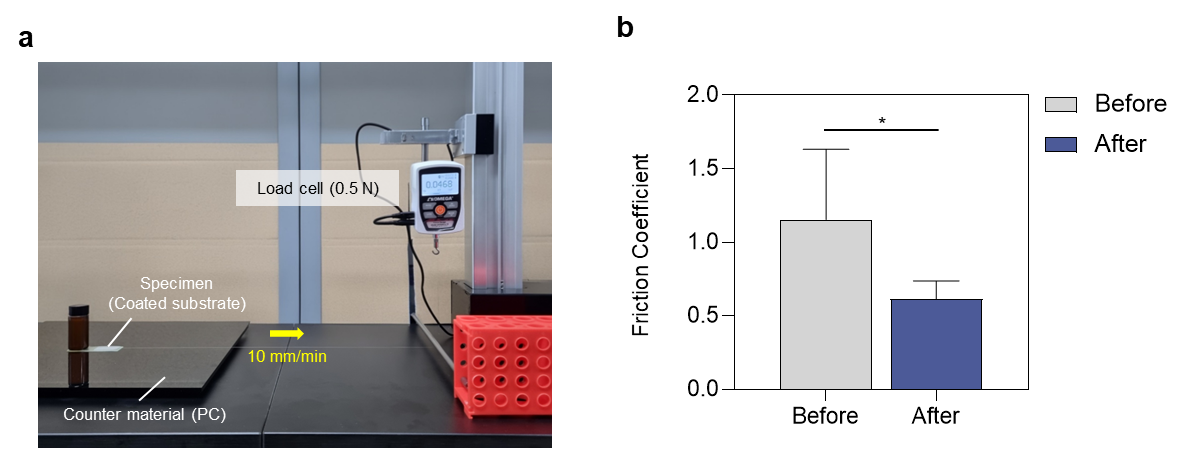


Supplementary Fig. 5

Standard-aligned interfacial friction measurement before and after coating. **a**, Experimental setup for interfacial friction measurement. A custom fixture was used to reproduce the coating-counterface contact geometry, while tangential force was measured using a calibrated commercial load cell/force gauge. The test was performed at a sliding speed of 10 mm min^−1^ against a polycarbonate counter material. **b**, Kinetic friction coefficient of the substrate before and after coating, calculated from the steady-state tangential force normalized by the applied normal load (*n* = 5). Data are shown as mean ± s.d., unpaired t-test (**p* < 0.05, ***p* < 0.01, ****p* < 0.001, *****p* < 0.0001, ns, not significant).


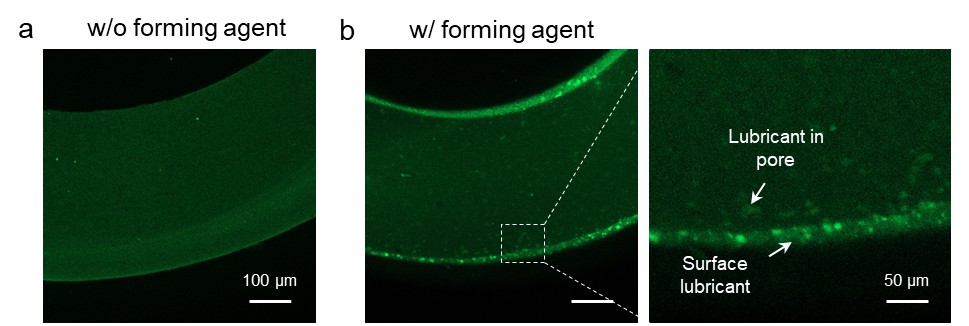


Supplementary Fig. 6

Cross-sectional confocal fluorescence images of stent materials. **a** Sample fabricated without foaming agent, showing a dense internal morphology. **b** Sample fabricated with foaming agent, exhibiting porous microstructure.


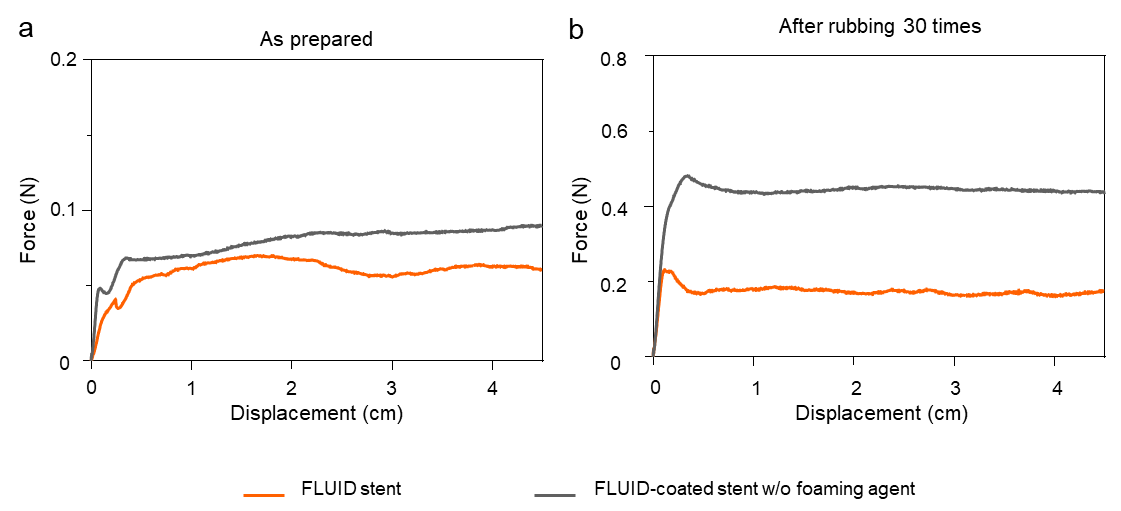


Supplementary Fig. 7

Frictional force–displacement curves of FLUID stents and FLUID-coated stent w/o foaming agent. **a** as prepared and **b** after rubbing. (*n*=3)


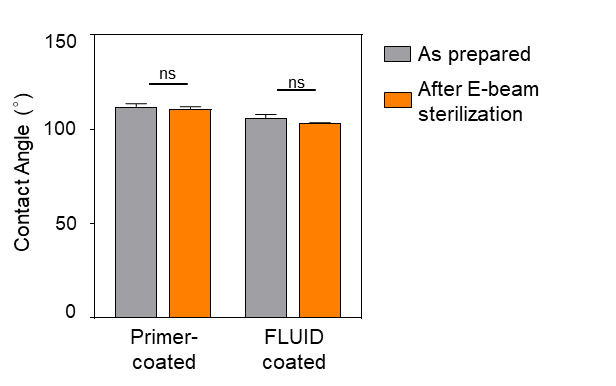


Supplementary Fig. 8

Water contact angle of FLUID-coated substrates before and after electron-beam sterilization (*n* = 3). Data are shown as mean ± s.d., unpaired t-test (**p* < 0.05, ***p* < 0.01, ****p* < 0.001, *****p* < 0.0001, ns, not significant).


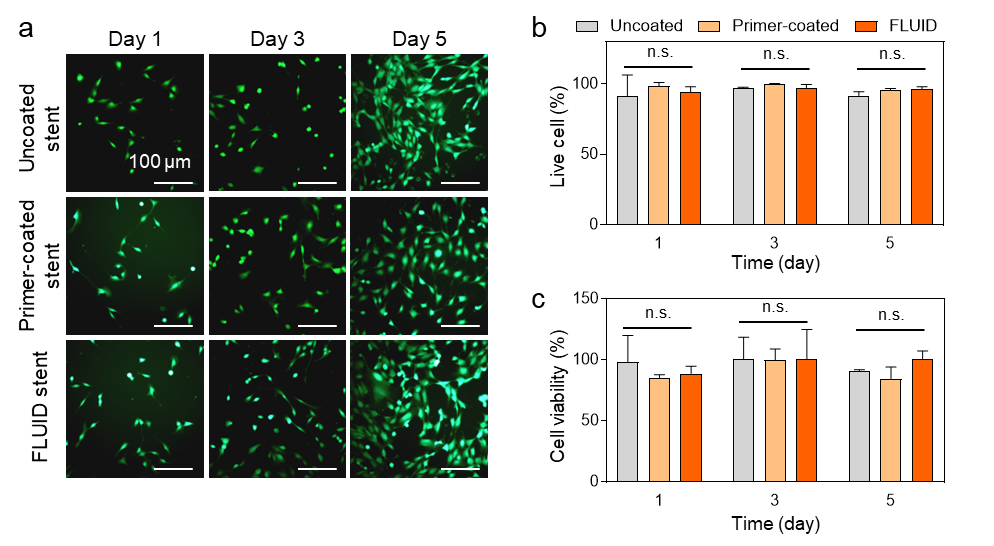


Supplementary Fig. 9

**a,b** Fluorescence microscopic images of NIH3T3 cells of uncoated stent and FLUID stent and statistical analysis (*n* = 3) (Scale bars: 100 µm). **c** Relative cell viability measurement with CCK-8 kit (*n* = 5). Data are shown as mean ± s.d., unpaired t-test (**p* < 0.05, ***p* < 0.01, ****p* < 0.001, *****p* < 0.0001, ns, not significant).


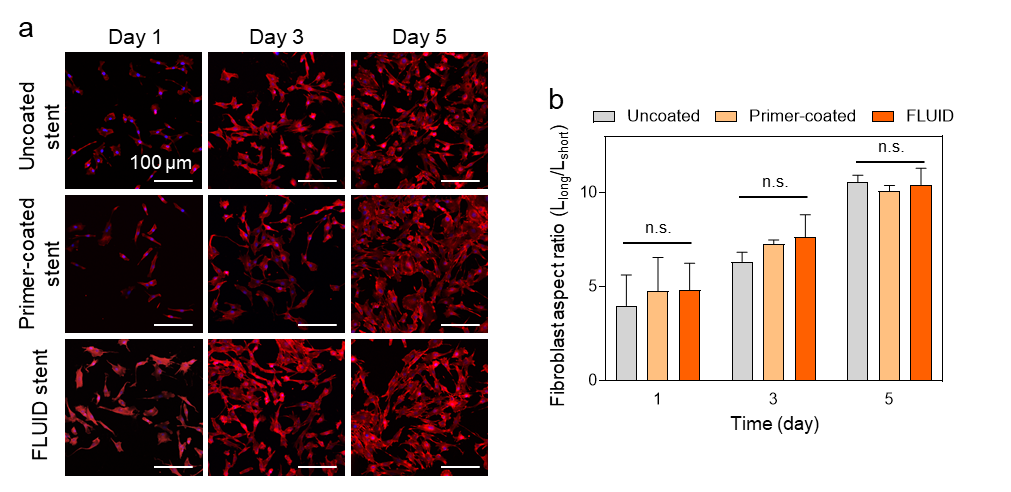


Supplementary Fig. 10

**a** Fluorescence microscopic images of NIH3T3 cells of uncoated PDMS and coated PDMS, and **b** statistical analysis (*n* = 3). Data are shown as mean ± s.d., unpaired t-test (**p* < 0.05, ***p* < 0.01, ****p* < 0.001, *****p* < 0.0001, ns, not significant).


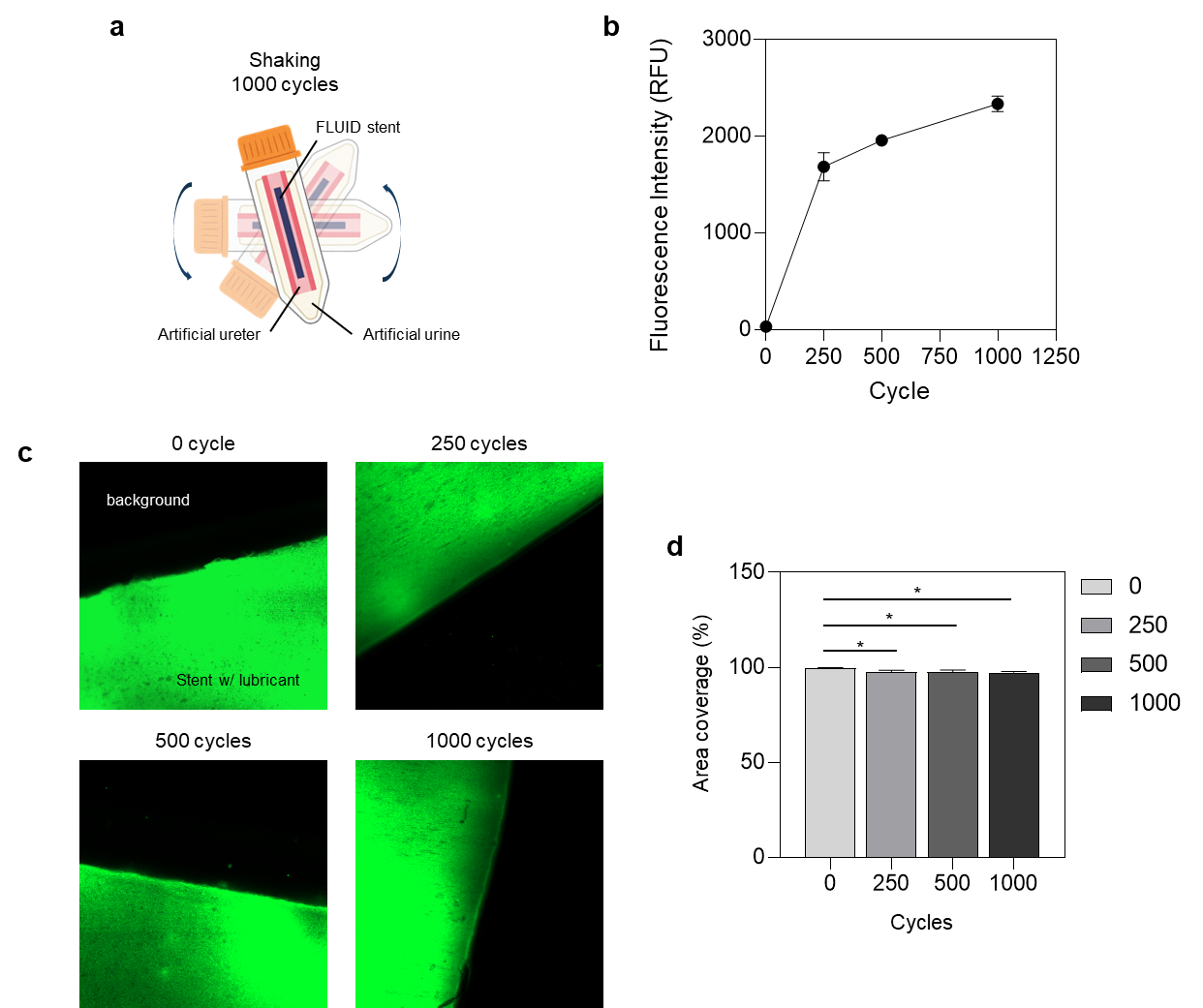


Supplementary Fig. 11

Evaluation of lubricant retention on the stent surface under dynamic conditions. **a** Schematic illustration of the experimental setup, where the stent was placed inside an artificial ureter and subjected to repeated agitation in artificial urine. **b** Fluorescence intensity measured from the surrounding artificial urine (*n* = 3). **c** Representative fluorescence images of the stent surface after shaking cycles. **d** Quantitative analysis of fluorescence coverage on the stent surface after shaking cycles (*n* = 3). Data are presented as mean ± standard deviation. Statistical significance was assessed using one-way analysis of variance followed by Dunnett’s multiple comparisons test. (**p* < 0.05, ***p* < 0.01, ****p* < 0.001, *****p* < 0.0001, ns, not significant).


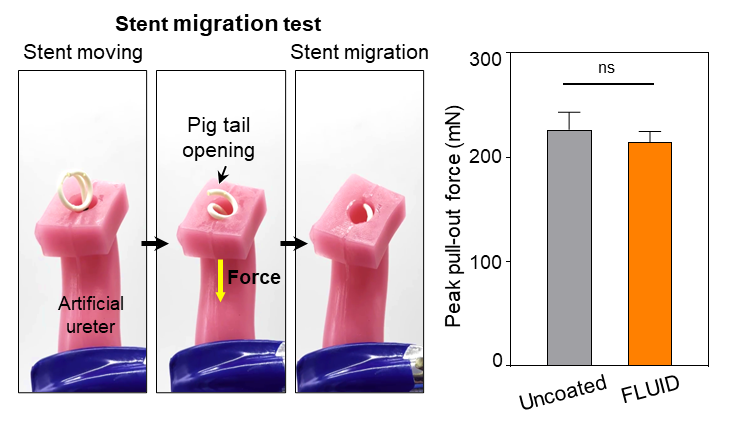


Supplementary Fig. 12

Stent migration test in an artificial ureter model with peak pull-out force measurement (*n* = 3). Data are shown as mean ± s.d., unpaired t-test (**p* < 0.05, ***p* < 0.01, ****p* < 0.001, *****p* < 0.0001, ns, not significant).


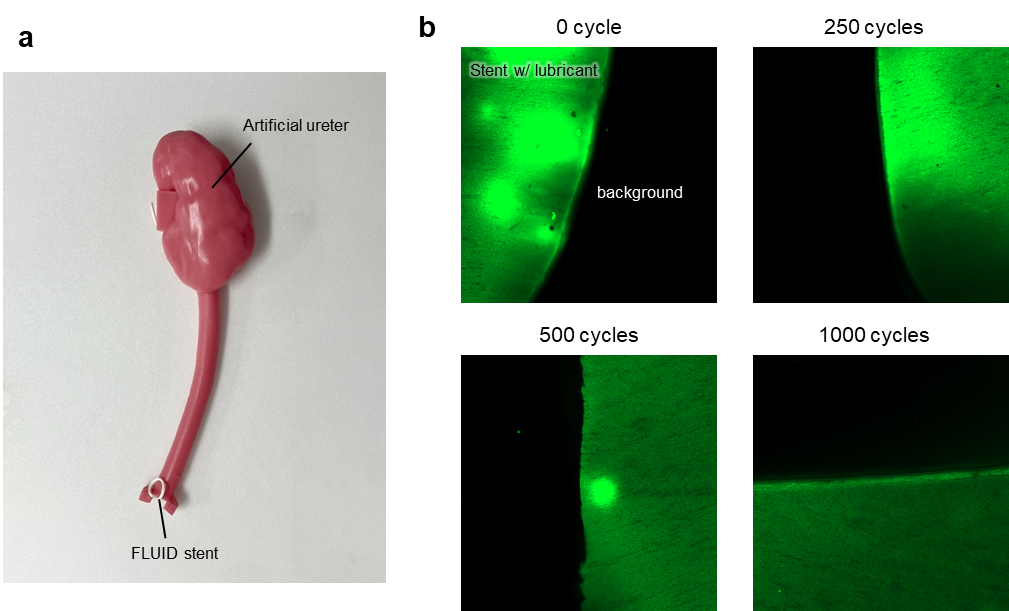


Supplementary Fig. 13

Lubricant retention on the stent surface after repeated physical friction. (a) Photograph of the artificial ureter model used for the friction test with the FLUID stent inserted. (b) Representative fluorescence images of the stent surface labeled with coumarin 6 before and after repeated friction cycles (0, 250, 500, and 1000 cycles).


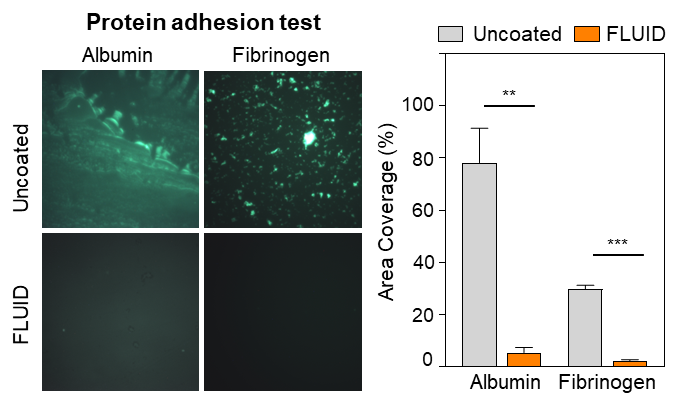


Supplementary Fig. 14

Protein adhesion test with fluorescence images and quantification of albumin and fibrinogen adsorption on uncoated and FLUID substrates (*n* = 3). Data are shown as mean ± s.d., unpaired t-test (**p* < 0.05, ***p* < 0.01, ****p* < 0.001, *****p* < 0.0001, ns, not significant).


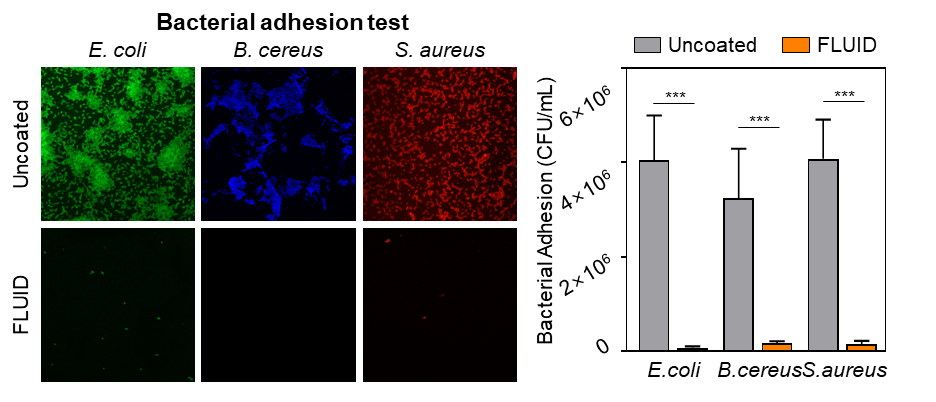


Supplementary Fig. 15

Fluorescence images and quantification of *E. coli*, *B. cereus*, and *S. aureus* adhesion on non-coated and coated stents (*n* = 3). Data are shown as mean ± s.d., unpaired t-test (**p* < 0.05, ***p* < 0.01, ****p* < 0.001, *****p* < 0.0001, ns, not significant).


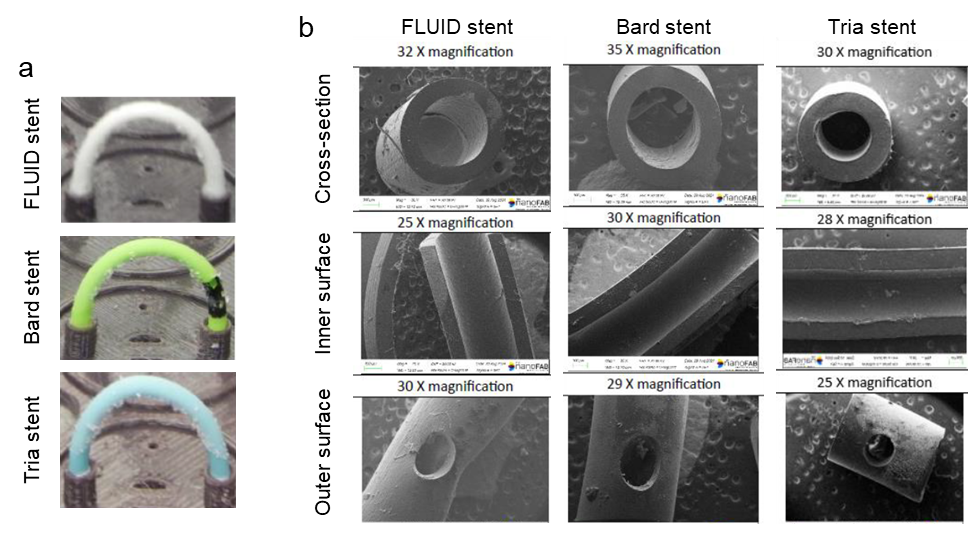


Supplementary Fig. 16

**a** Optical images and **b** SEM images after 2 weeks *in vitro* encrustation test in sterile urine.


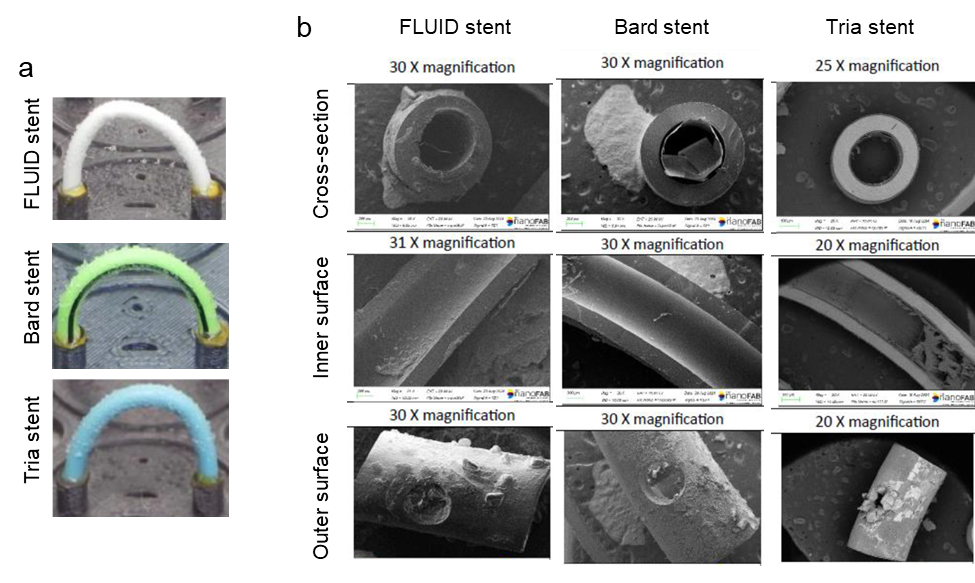


Supplementary Fig. 17

**a** Optical images and **b** SEM images after 2 weeks *in vitro* encrustation test in spiked urine.

Supplementary Table 1.

Benchmarking of FLUID against representative state-of-the-art anti-fouling coatings.

| **Platform** | **Substrate / Geometry** | **Coating method** | **Lubricant type** | **Durability** | **In vivo validation** |
| --- | --- | --- | --- | --- | --- |
| SLIPS  [1] | Planar nanostructured substrate | Lithography / etching + infusion | Perfluorinated oil | Surface-confined reservoir; depletion under shear | Not demonstrated |
| TLP  [2] | Planar / tubing, medical devices | Tethered fluorosilane + perfluorocarbon | Perfluorocarbon | Hours to days under blood flow | Short-term vascular / extracorporeal |
| Zwitterionic coatings  [3] | Planar / tubular | Graft-from polymerization | Hydrated polymer brush | Susceptible to oxidative / mechanical damage | Limited short-term studies |
| Peptide-modified stent  [4] | Ureteral stent | Mussel-inspired click chemistry | Antibacterial peptide | ~2 weeks *in vivo* | Rat model |
| Hydrogel / PVP coated stent  [5] | Ureteral stent | Solvent casting / graft polymerization | Hydrophilic polymer (hydrated) | Swelling / delamination under abrasion | Short-term only |
| FLUID (this work) | Full-length double-J ureteral stent | Dip-coating with wall-distributed microporous reservoir | Medical-grade silicone oil (PFAS-free) | Sustained retention >4 weeks orbital shaking; 1000 rubbing cycles | Porcine model, 4 and 8 weeks |

Supplementary Table 2.

Porosity values of TPU stents measured by mercury intrusion porosimetry. Total, interparticle, and intraparticle porosity are summarized for each sample.

| **Sample** | **Total porosity (%)** | **Interparticle (%)** | **Intraparticle (%)** |
| --- | --- | --- | --- |
| S1 | 5.95 | 2.17 | 3.79 |
| S2 | 9.19 | 2.91 | 6.28 |
| S3 | 3.57 | 3.14 | 0.43 |
| **Average** | **6.24** | - | - |

Supplementary Table 3.

XPS peaks indicating the atomic concentrations of various components of the uncoated stent and the FLUID stent. Based on the increased Si ratio, interface primer coating is well-formed on the stent.

| Peak | | C 1s | N 1s | O 1s | Si 2p |
| --- | --- | --- | --- | --- | --- |
| Atomic  Concentration  (%) | FLUID stent | 43.2 | 0.4 | 24.6 | 31.8 |
|  | Uncoated stent | 75.6 | 2.4 | 19.3 | 2.7 |

Supplementary Table 4.

Summary of biological evaluation tests conducted in accordance with ISO 10993 standards for medical devices, including cytotoxicity, pyrogenicity, intracutaneous reactivity, and acute systemic toxicity.

| **ISO Standard** | **Test Item** | \| **Test Subject** \| \| --- \| | \| **Result** \| \| --- \| | \| **Evaluation** \| \| --- \| |
| --- | --- | --- | --- | --- | --- | --- | --- |
| ISO 10993-5 | Cytotoxicity test | L-292 cell | Grade 0  (no cytotoxicity observed) | Compliant |
| ISO 10993-11 | Pyrogenicity test | New Zealand White Rabbit | Change in body temperature  < +0.5 °C | Compliant |
| ISO 10993-23 | Intracutaneous reactivity test | New Zealand White Rabbit | Final irritation score:  polar extract 0.0,  non-polar extract 0.0  (non-irritant) | Compliant |
| ISO 10993-11 | Acute systemic toxicity test | Mouse | No abnormal clinical signs, deaths, body-weight changes, or gross pathological findings | Compliant |

Supplementary Table 5.

Histopathological scoring of urothelial tissues (*n* = 3).


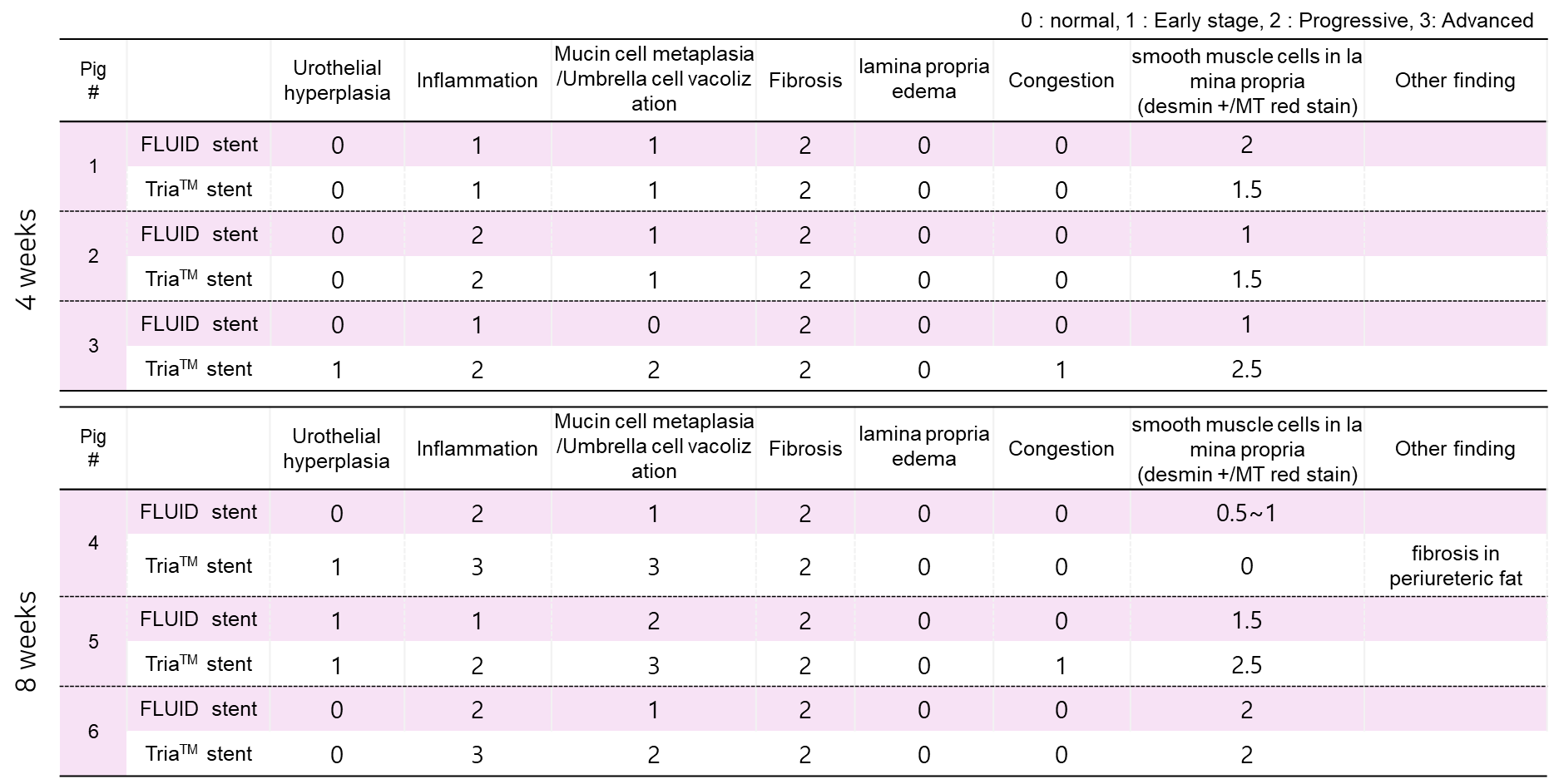


Supplementary Movie 1.

The anti-adhesion tests of Uncoated and FLUID-coated stent.

Reference

1. Wong, T.-S., Kang, S. H., Tang, S. K., Smythe, E. J., Hatton, B. D., Grinthal, A., Aizenberg, J., Bioinspired self-repairing slippery surfaces with pressure-stable omniphobicity, Nature, 477 (2011) 443, <https://doi.org/10.1038/nature10447>

2. Leslie, D. C., Waterhouse, A., Berthet, J. B., Valentin, T. M., Watters, A. L., Jain, A., Kim, P., Hatton, B. D., Nedder, A., Donovan, K., A bioinspired omniphobic surface coating on medical devices prevents thrombosis and biofouling, Nature biotechnology, 32 (2014) 1134, <https://doi.org/10.1038/nbt.3020>

3. Jiang, S., and Cao, Z., Ultralow‐fouling, functionalizable, and hydrolyzable zwitterionic materials and their derivatives for biological applications, Advanced materials, 22 (2010) 920, <https://doi.org/10.1002/adma.200901407>

4. Yao, Q., Zhang, J., Pan, G., Chen, B., Mussel-inspired clickable antibacterial peptide coating on ureteral stents for encrustation prevention, ACS Applied Materials & Interfaces, 14 (2022) 36473, <https://doi.org/10.1021/acsami.2c09448>

5. Tang, H., Wu, D., Liu, Z., Liu, X., Yuan, H., Jin, X., Gao, S., Chen, G., Polyvinylpyrrolidone hydrogel coating for ureteral stent: Safety and performance evaluation, Bio-Medical Materials and Engineering, 35 (2024) 205, <https://doi.org/10.3233/BME-230179>
